# Research Progress on Intrinsically Conductive Polymers and Conductive Polymer-Based Composites for Electromagnetic Shielding

**DOI:** 10.3390/molecules28227647

**Published:** 2023-11-17

**Authors:** Yuzhen Zhao, Chaonian Li, Tingting Lang, Jianjing Gao, Huimin Zhang, Yang Zhao, Zhun Guo, Zongcheng Miao

**Affiliations:** 1 Technological Institute of Materials & Energy Science (TIMES), Xi’an Key Laboratory of Advanced Photo-Electronics Materials and Energy Conversion Device, School of Electronic Information, Xijing University, Xi’an 710123, China; zyz19870226@163.com (Y.Z.); lichaonian0513@126.com (C.L.); langtingting516@163.com (T.L.); gaojianjing.ch@163.com (J.G.); grance0127@126.com (H.Z.); zhaoyang@xijing.edu.cn (Y.Z.); guozhun1987@163.com (Z.G.); 2 School of Artificial Intelligence, Optics and Electronics (iOPEN), Northwestern Polytechnical University, Xi’an 710072, China

**Keywords:** electromagnetic shielding, electromagnetic interference, intrinsically conductive polymers, conductive polymer-based composites

## Abstract

Electromagnetic shielding materials are special materials that can effectively absorb and shield electromagnetic waves and protect electronic devices and electronic circuits from interference and damage by electromagnetic radiation. This paper presents the research progress of intrinsically conductive polymer materials and conductive polymer-based composites for electromagnetic shielding as well as an introduction to lightweight polymer composites with multicomponent systems. These materials have excellent electromagnetic interference shielding properties and have the advantages of electromagnetic wave absorption and higher electromagnetic shielding effectiveness compared with conventional electromagnetic shielding materials, but these materials still have their own shortcomings. Finally, the paper also discusses the future opportunities and challenges of intrinsically conductive polymers and composites containing a conductive polymer matrix for electromagnetic shielding applications.

## 1. Introduction

With the rapid development of science and technology and the widespread use of electronic devices, electromagnetic interference has become an important issue. Electromagnetic radiation can affect or damage the normal operation of electronic devices/circuits, leading to data loss, and may also affect biological processes including human health. In light of these important issues, researchers have been looking for new and better EMI shielding materials. At present, the main research on electromagnetic shielding materials has focused on the following aspects: first, optimizing the shielding characteristics of materials, especially high frequency and large electromagnetic shielding characteristics; second, to improve their mechanical properties and increase their service life; third, to achieve the goal of sustainable development, mainly the development of renewable, biodegradable electromagnetic wave shielding materials; fourth, the electromagnetic shielding materials and other materials for composites, so that it has a better overall performance; fifth is to make electromagnetic shielding materials with low density and are lightweight [1].

Compared with traditional metallic electromagnetic shielding materials, ICP (intrinsically conducting polymers) and CPC (conductive polymer-based composites) have advantages in terms of mechanical properties, corrosion resistance, and weight. In addition, when polymers are compounded with nanostructured materials, they do not affect the electromagnetic interference (EMI) shielding performance at the same time. Not only can it prevent its agglomeration, but also improve its physical properties [2,3,4,5,6]. This paper presents a comprehensive analysis of electromagnetic interference (EMI) mechanisms in advanced polymeric materials including the interaction between polymers and conductive fillers, the influence of the arrangement pattern and concentration of fillers in the polymer matrix on the shielding effectiveness, and the permeation thresholds of different composites. Furthermore, it discusses the latest research on lightweight polymer composites and multicomponent systems used for EMI shielding, which are based on ICP and CPC. The article concludes by forecasting the future development trends for EMI shielding materials.

## 2. Basic Principles of Electromagnetic Shielding and Measurement of Shielding Effectiveness

The shielding performance of electromagnetic shielding materials is mainly affected by three main factors: their thickness, electrical conductivity, and permeability. Generally, thicker shielding materials and higher electrical conductivity are better for shielding. In addition, the electromagnetic shielding effect is different at different frequencies and wavelengths of electromagnetic waves. Therefore, there is a need to choose the right EMI shielding material for the application scenario.

### 2.1. EMI Shielding Effectiveness

The ability of electromagnetic shielding materials to weaken electromagnetic signals is defined by the electromagnetic shielding effectiveness (EMI SE), expressed as Equations (1)–(3).

(1) $$SE_{p}=10\log(P_{in}/P_{out})$$

(2) $$SE_{E}=20\log(E_{in}/E_{out})$$

(3) $$SE_{H}=20\log(H_{in}/H_{out})$$

(4) $$SE_{T}=SE_{E}=SE_{P}=SE_{H}$$

EMI SE is measured in decibels (dB). Electromagnetic waves consist of electric (E) and magnetic (H) fields that are perpendicular to each other and propagate perpendicular to the plane that contains them. The strength of these fields is represented by the plane wave strength (P), electric field strength (E), and magnetic field strength (H), respectively. The subscript “in” indicates the field strength of the transmission incident to the electromagnetic shielding material, and the subscript “out” indicates the field strength of the transmission through the electromagnetic shielding material. The wave impedance is defined as the ratio of the electric field strength to magnetic field strength and varies depending on the frequency and energy of the electromagnetic wave. The measurement area is divided into far-field and near-field regions according to the distance (r) between the EMI shielding material and the electromagnetic wave source. E/H is called the electromagnetic impedance. In the far-field region (i.e., when r > λ/2π), the value of the electromagnetic impedance is the same as the intrinsic impedance of free space Z_0_, which is 377 Ω. Therefore, in the far-field region, SE_E_ = SE_H_, there is a plane wave. However, when r < λ/2π, at this time SE_E_ ≠ SE_H_, and in the near-field region, the electromagnetic impedance is different from the intrinsic impedance Z_0_ of free space. In this region, if an electromagnetic impedance E/H < Z_0_ = 377 Ω, it indicates that it is dominated by the magnetic fields, and vice versa. In addition, when r ≈ λ/2π, it is the transition point between the far-field and near-field regions [2,7,8,9].

### 2.2. Electromagnetic Shielding Mechanism

Shielding materials mainly reduce electromagnetic waves through the absorption and reflection of electromagnetic waves. The reflection mechanism causes radiation to bounce back from the shielding material. However, the reflected radiation may cause harm to the environment or to the people present. Therefore, the absorption mechanism is preferred for shielding from the safety point of view.

**Figure 1:**
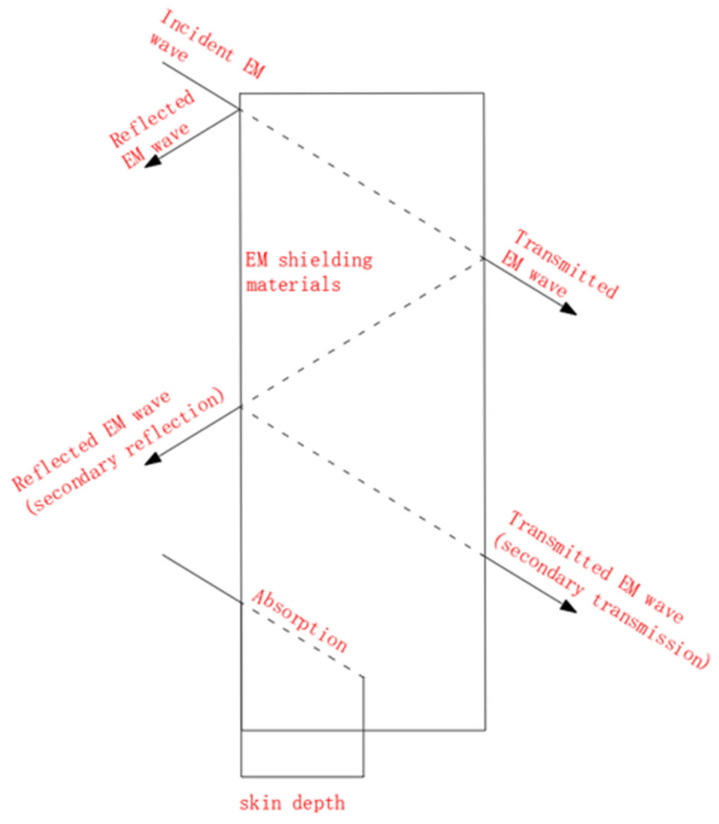
Schematic diagram of skin depth and electromagnetic shielding.

EMI shielding materials exhibit the total EMI SE through absorption, reflection, and multiple internal reflections. Incident electromagnetic waves are reflected and propagated within the material due to the difference in inherent impedance between the material and the wave propagation medium. The intensity of the transmitted and reflected waves is related to the impedance of the material and the medium. The transmitted wave’s intensity exponentially decreases as it propagates within the material until it reaches a depth known as the skin depth (δ), where its intensity becomes 1/e (e is the Euler number, 1/e = 0.37). If the wave reaches another surface of the material, a portion of it will be reflected several times and another portion will penetrate the material [10], as shown in Figure 1.

A better skin depth can be obtained when σ is much larger than $2\pi \omega \varepsilon_{o}$, and the skin depth is calculated by Equation (4)

(5) $$\delta =\sqrt{\frac{1}{\pi \omega \mu \sigma }}$$

Here, the frequency (ω), the relative permeability of the shielding material (μ), the electrical conductivity of the shielding material (σ), and the permittivity of free space ($\varepsilon_{o}$ = 8.854 × 10^12^ F/m) [11].

(6) $$SE_{T}=-10\log(T)$$

(7) $$SE_{R}=-10\log(1-R)$$

(8) $$SE_{A}=SE_{T}-SE_{R}-SE_{M}$$

The equation involves two coefficients: the transmission coefficient (T) and the reflection coefficient (R). T represents the fraction of the input power that penetrates the sample, while R represents the fraction of the input power that bounces off the sample surface. Typically, if the total shielding effectiveness ($SE_{T}$) is greater than 15 dB, the shielding effect of multiple reflections inside the material ($SE_{M}$) is often ignored because it is too weak.

(9) $$SE_{A}=131.4t\sqrt{\mu_{r}\sigma_{r}f}=131.4\sqrt{f\mu \sigma /\left(\mu_{0}\sigma_{Cu}\right)}=8.68t\sqrt{\pi f\mu \sigma }$$

where t is the thickness of the specimen (in m), f is the frequency of the magnetic field applied to the specimen (in Hz), $\mu_{r}$ is the relative magnetic permeability of the specimen compared to copper, and $\sigma_{r}$ is the electrical conductivity compared to copper. Table 1 presents the σ and μ of other materials relative to copper [12]. σ is the electrical conductivity and μ is the magnetic permeability, where $\mu =\mu_{0}*\mu_{r}$ and $\mu_{0}$ = 4π × 10^−7^ H/m. The equation is for the case of conductive materials. Assuming that the magnetic permeability ($\mu_{r}$) and electrical conductivity ($\sigma_{r}$) remain constant with increasing frequency, the $SE_{A}$ increases with increasing frequency. In addition, $SE_{A}$ increases when both the magnetic permeability and electrical conductivity increase at a particular frequency.

The shielding mechanism of an electromagnetic shielding material can be understood by measuring its dielectric (relative complex permittivity, $\varepsilon_{r}={\varepsilon^{\prime }}_{r}-j{\varepsilon^{\prime \prime }}_{r}$) and magnetic (relative complex permeability, $\mu_{r}={\mu^{\prime }}_{r}-j{\mu^{\prime \prime }}_{r}$) properties. The solid parts ${\varepsilon^{\prime }}_{r}$, ${\mu^{\prime }}_{r}$ represent the charge storage and magnetic storage of electromagnetic waves, respectively, and the imaginary parts ${\varepsilon^{\prime \prime }}_{r}$, ${\mu^{\prime \prime }}_{r}$ represent the dielectric and magnetic losses during the interaction of the shielding material with electromagnetic waves, respectively. The amount of loss can be calculated from the tangent values of dielectric loss ($\tan\delta_{\varepsilon }=\frac{{\varepsilon^{\prime \prime }}_{r}}{{\varepsilon^{\prime }}_{r}}$) and magnetic loss ($\tan\delta_{\mu }=\frac{{\mu^{\prime \prime }}_{r}}{{\mu^{\prime }}_{r}}$) [2,13].

(10) $$Z_{in}=Z_{0}\sqrt{\frac{\mu_{r}}{\varepsilon }}\tanh\left[j\left(\frac{2\pi fd}{C}\sqrt{\frac{\varepsilon_{r}}{\mu_{r}}}\right)\right]$$

(11) $$RL=20\log\left|\frac{\left(Z_{in}-Z_{0}\right)}{Z_{in}+Z_{0}}\right|$$

(12) $$Z=\left|\frac{Z_{in}}{Z_{0}}\right|$$

$Z_{in}$ is the input impedance of the EMI shielding material.

When $\left|Z_{0}\approx Z_{in}\right|$ (i.e., Z=1), it means that the two reach the best impedance matching. At this time, the electromagnetic wave reflected from the surface of the shielding material decreases, while the electromagnetic wave entering the interior increases, and the absolute value of RL can also reach the peak. At this point, one can let the electromagnetic wave as much as possible into the material inside by converting the electromagnetic wave to other forms of the purpose of its attenuation. However, when the impedance matching performance is poor, a large number of electromagnetic waves are reflected or scattered by the material, resulting in a significant decrease in the absolute value of the reflection loss.

The synergistic effect between the dielectric and magnetic losses helps to enhance the wave absorbing material to form a good balance between the impedance matching and attenuation mechanisms, so the prerequisite for high performance electromagnetic wave absorption is that the absorbing material has good impedance matching and attenuation mechanisms. The condition to achieve full impedance matching is $\mu^{\prime }/\varepsilon^{\prime }=1$. For most materials, $\mu^{\prime }$ is smaller than $\varepsilon^{\prime }$. The higher the values of $\varepsilon^{\prime \prime }$ and $\mu^{\prime \prime }$, the larger the attenuation constant and hence the larger the attenuation. However, a high value of $\mu^{\prime \prime }$ produces a significant dielectric heating effect, which may lead to overheating of the material and consequent failure. Therefore, when selecting electromagnetic shielding materials in practical applications, in order to achieve the best shielding effect, it is necessary to comprehensively consider the influence of various aspects.

### 2.3. Measurement of Shielding Effectiveness

The shielded box method, shielded room method, coaxial transmission line test, and open field or free space method are the four most common methods used to study the electromagnetic interference shielding properties of materials.

**Figure 2:**
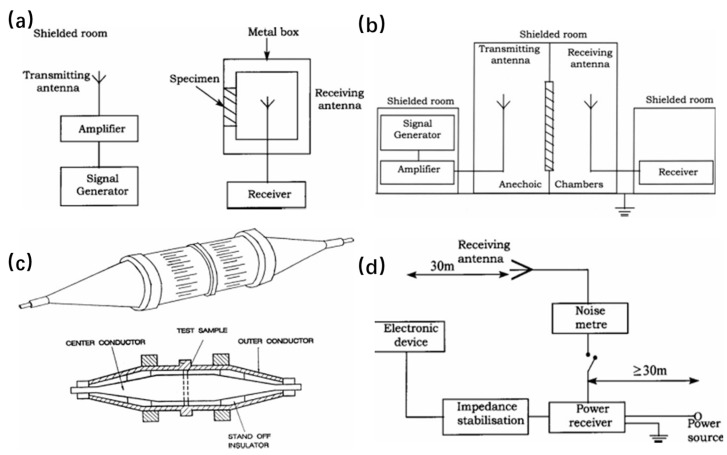
(**a**) Shielded box method; (**b**) shielded room method; (**c**) coaxial transmission line test method; (**d**) open field or free space method [14].

The shielding box method utilizes a transmitting antenna outside the shielded room and a receiving antenna inside the shielded room to test the attenuation of transmitted waves after the electromagnetic wave passes through the material to calculate the shielding effectiveness. However, due to the influence of the experimental environment, the results obtained in different laboratories are not comparable, and can only measure a very limited frequency range, as shown in Figure 2a.

The shielding room method, which was developed to overcome the shortcomings of the shielding box method, has a similar principle to the shielding box method. Its receiving and transmitting antennas and electromagnetic source generators are isolated separately, eliminating the impact of environmental interference and improving the reliability of the data, but the structure is relatively complex, as shown in Figure 2b.

In the coaxial transmission line test method, the basic principle of the coaxial test method is installed in the coaxial sample frame by the material to be tested; in the test, the signal is transmitted through the coaxial cable coupled with the attenuator, then the signal is transmitted through the material to be tested, and then through the network analyzer to measure the strength of the transmitted signal, in order to assess the shielding performance of the material. The coaxial transmission line test method not only requires a relatively small sample, but also addresses the shielding effects caused by absorption, transmission and reflection, respectively, as shown in Figure 2c.

The open-field or free-space method is used for the actual shielding effectiveness of electronic systems. The test does not directly determine the properties of a particular material and results can vary widely. As shown in Figure 2d, the test is performed by separating the equipment from the receiving antenna by 30 m and recording the radiated emission, while at the same time recording the conducted radiation transmitted through the cable [14].

## 3. Intrinsically Conductive Polymers (ICP)

ICP is a polymer with excellent electrical conductivity. Such polymers can possess metal-like conductivity or have semiconducting properties. When the polymer structure has extended conjugated double bonds, the off-domain π-bonded electrons are not bound by atoms and can move freely in the polymerization chain, and after doping, the electrons can be removed to generate holes, or electrons can be added so that the electrons or holes can move freely in the molecule chain to form conductive molecules. Therefore, ICP materials themselves can be used as electromagnetic shielding materials, but the large number of π-electron delocalization in the ICP conjugate structure will reduce the processing performance of ICP materials. In addition, the mechanical and electrical properties of ICP materials are affected by their own tendency to expand, contract, crack, or soften. Common conducting polymers include polyaniline, polypyrrole, polythiophene, and poly (p-phenylene vinylene) as well as their derivatives. Although ICP materials have excellent electrical conductivity, pure ICP is rarely used as a shielding material due to its low processing and mechanical properties [15].

Nonetheless, the characteristics of ICP materials can be enhanced through the integration of secondary materials. These auxiliary materials improve the shielding properties of ICP materials while not largely adversely affecting the ICP materials. As a result, the EMI SE and ${\varepsilon^{\prime \prime }}_{r}$ values of ICP composites are higher than those of pure ICP, which is attributed to the greatly enhanced interfacial polarization of the large area of ICP composites and the inconsistent dielectric properties between ICP and second-phase materials.

### 3.1. Factors Affecting the Performance of ICP Materials

Material preparation methods and different dopants: ICP can be doped with small molecules to increase its electrical conductivity. The doping level can be controlled by adjusting the concentration of the dopant. Sodium dodecyl sulfate doping limits the bias dependence of carrier interchain jumps in ICP materials, thus improving the conductivity and EMI SE [16]. In addition, changing the chemical and physical properties of the material by adding acid can also affect its EMI SE. A study showed that phosphoric acid (H_3_PO_4_) led to the nanofibrous structure of polyaniline (PANI), while HCl and L(-)-camphorsulfonic acid (CSA) led to the production of the holothurian-like structure of PANI. CSA-doped PANI had the highest electrical conductivity due to the presence of hydroxyl groups as well as morphological effects that greatly facilitated electron dispersion [17]. He et al. experimentally demonstrated that composites could be prepared from wood extracted from sapele bark by compounding with phosphoric acid. When the concentration of H_3_PO_4_ gradually increased, the conductivity of the composites increased accordingly. However, when the addition of H_3_PO_4_ exceeded 0.6 M, the conductivity of the composites decreased. When the concentration of phosphoric acid was 0.6 M, the σ value of the prepared wood/PANI composite was 9.53 × 10^−3^ S·cm^−1^, and when the electromagnetic wave frequency was within 10–1.5 GHz, its EMI SE was 45–60 dB [18]. In addition, Ag-polypyrrole(PPy)-MWCNTs (multi-walled carbon nanotubes) composites prepared by UV reduction and chemical reduction were compared in terms of electrical resistance and EMI SE. Composites prepared by UV reduction contained higher (57%) and more uniformly distributed silver nanoparticles at the interface compared to those produced by chemical reduction (40%), thus exhibiting higher resistance and EMI SE [19].

Processing and reaction conditions: Processing conditions such as temperature and pressure can affect the morphology and conductivity properties of ICP films. Wan et al. conducted an experiment in which they investigated the effect of reaction temperature on the electrical conductivity of bacterial cellulose/graphene/polyaniline (BC/GN/PANI) composites. They observed that the σ of the composites gradually decreased from 0.82 S·cm^−1^ to 0.74 S·cm^−1^ when temperature of the reaction was gradually increased from 0 °C to 25 °C, while the σ increased from 8.5 × 10^−5^ S·cm^−1^ when the reaction time was increased from 2 to 10 h up to 1 S·cm^−1^ [20]. As the temperature increases, the tendency of the conductive polymer to agglomerate increases, leading to a decrease in conductivity. Conversely, when the temperature decreases, the amount of conductive polymer increases with time, leading to an increase in its conductivity.

In addition to the above factors, the structure of the polymer, molecular weight, and film thickness can also affect the shielding effectiveness of ICP. Therefore, when using ICP as an electromagnetic shielding material, it is necessary to consider all of the above factors and choose the most suitable shielding material.

### 3.2. ICP-Based EMI Shielding Materials

Carbon nanotubes, graphene, and metal nanoparticles/fibers are among the most commonly used substances as ICP additives because of their unique structure and excellent electrical conductivity, which can greatly enhance the shielding performance of ICP [21]. Carbon fibers (CNFs) are ideal electromagnetic shielding materials. Qiao et al. prepared a high performance porous CNF paper by the wet papermaking process and the EMI SE of this material was 60 dB at the X-band (8–12 GHz). In addition, they fabricated porous Ni@CNF paper by chemical plating. The 3D network structure of the Ni@CNF paper achieved an electrical conductivity of 400 S·cm^−1^ at a thickness of only 0.36 mm, and the EMI SE of the Ni@CNF paper reached 120 dB, also at the X-band. In addition, the Ni@CNF paper had good permeability, corrosion resistance, and mechanical properties [22].

A total of 20 wt% of silver nanowires (AgNWs) were added to PPy to achieve a flexible EMI shielding film. The conductivity of the PPy/AgNWs film was improved from 0.02 S·cm^−1^, which is the conductivity of pure PPy, to 62.7 S·cm^−1^. As a result, the shielding effectiveness of the film at the X-band increased to 22.4 dB [23].

**Figure 3:**
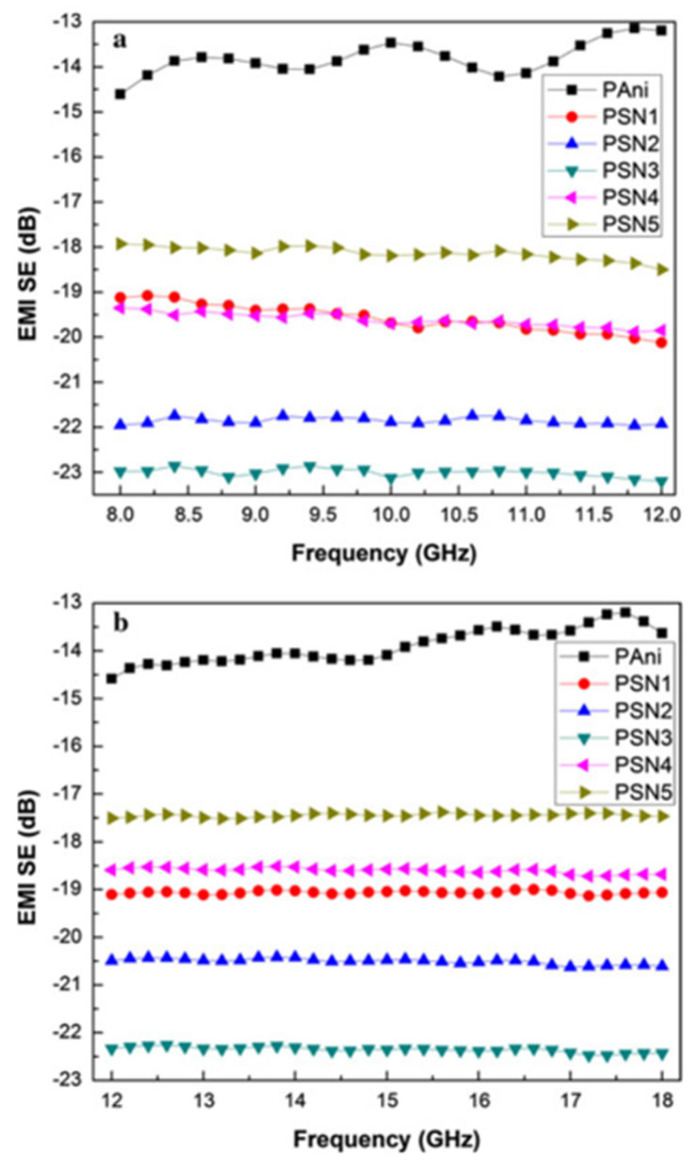
The EMI SE of different PANI/SnO composites at the (**a**) 8–12 GHz and (**b**) 12–18 GHz frequency bands [24].

**Figure 4:**
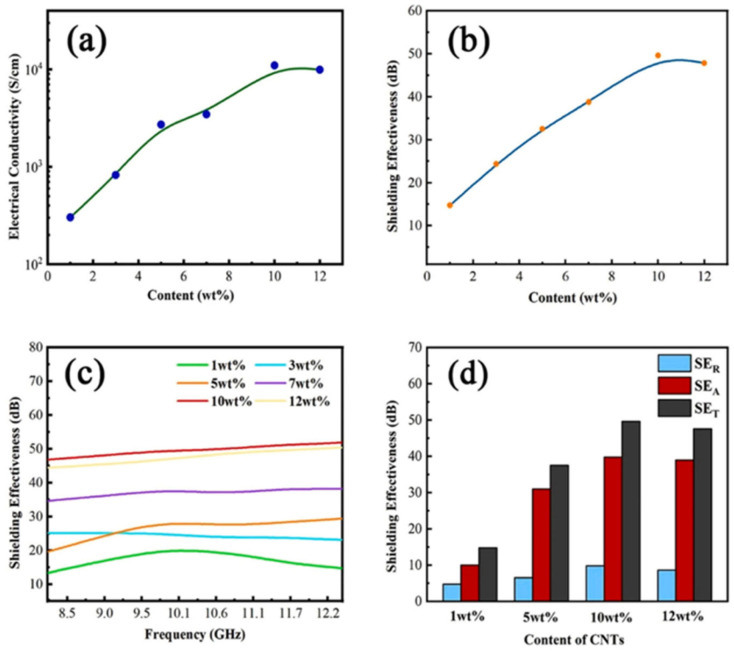
(**a**) Electrical conductivity of the composites. (**b**) EMI SE with varying CNT content. (**c**) X-band EMI SE of the composites. (**d**) SE_A_, SE_R_, and SE_T_ for different CNT contents [25].

However, metal nanoparticles can be affected by corrosion. Metal oxides are often used as a substitute for metal nanoparticles in the integration of ICP materials. The movement of charge carriers within the ICP backbone is hindered by the formation of modified phases at the interface by metal oxides, which in turn increases the capacitance of ICP composites and improves their dielectric constants. In addition, metal oxide fillers are not only corrosion resistant but are also lighter in mass [1]. The EMI SE of the PANI/Sb_2_O_3_ composites is 18 to 21 dB and 17.5 to 20.5 dB in the X-band and ku-band (12–18 GHz), respectively. It was further found that after doping PANI with different weight percentages of SnO, EMI SE was positively correlated with the amount of SnO until a critical concentration was reached. However, when the SnO loading concentration was further increased, the EMI SE started to decrease instead. This may be due to the fact that higher concentrations of SnO disrupt the continuity of the polymer chains, leading to a decrease in shielding effectiveness [24,25], as shown in Figure 3. Similarly, some researchers have found that the addition of excessive CNTs to the ICP composites they studied also resulted in a decrease in shielding effectiveness. Yang et al. obtained carbon nanotube (CNT)/bamboo fiber/HDPE composites (CNTs-BPC) by mixing CNTs, hydrophobic bamboo fibers, and high-density polyethylene (HDPE) and then hot pressing them at 10 MPa, 150 °C for 30 min. The CNTs built an excellent conductive network in this composite, where its conductivity reached 1.1 × 10^4^ S·cm^−1^ and the EMI SE in the X-band reached 49.6 dB when the CNT content was only 10 wt%. At 12 wt%, a slight decrease in conductivity was observed, as shown in Figure 4. This may be due to the disruption of the conductive network by the CNT aggregates [26]. Both articles found that an increase in dopant concentration resulted in a decrease in EMI SE, which the authors hypothesized was due to the fact that the increase in dopant concentration disrupted the continuity and conductivity of the polymer chains. The reason for this is not explicitly mentioned in the paper, but it does show that the dopant concentration is not completely positively correlated with EMI SE.

The addition of magnetic materials to the intrinsically conducting polymer (ICP) leads to a decrease in the conductivity of the composite due to the dissociation of the conducting chains in the ICP. Because intermittent non-magnetic ICP separates magnetic particles, the saturation magnetization and coercivity fields of the composite are reduced. However, the improvement in thermal stability compared to pure ICP is due to the suppression of polymer chain motion by magnetic particles at high temperatures, while the improvement in the EMI SE is due to the combined effect of the dielectric loss of ICP itself and the magnetic loss of magnetic material [27,28]. To enhance the electromagnetic shielding effectiveness, the electrical conductivity (σ) and magnetic permeability (μ) of ICP/magnetic material composites are two crucial factors.

**Figure 5:**
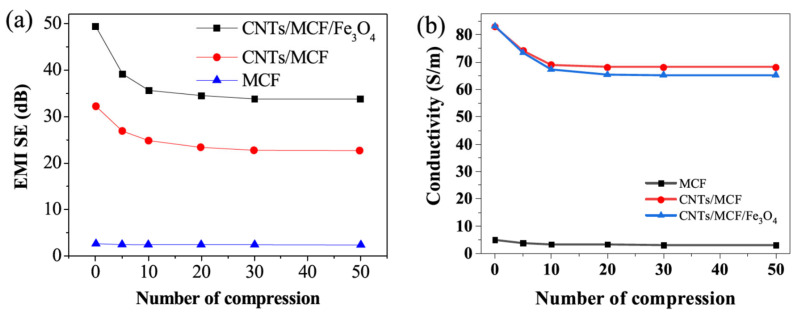
(**a**) EMI SE and (**b**) electrical conductivity after multiple folding of different materials [30].

Experimental results show that when embedded in an epoxy resin matrix with a thickness of 1 mm, the reflection loss (RL) of the composite changes depending on the weight percentage of polyaniline (PANI) or Fe_3_O_4_ in the composite. Specifically, the composite containing 15 wt% PANI and 10 wt% Fe_3_O_4_ exhibited a high RL of 42 dB at 16.3 GHz, while the composite containing 15% PANI and 25% Fe_3_O_4_ exhibited a slightly lower RL of 37.4 dB at 14.9 GHz. The composite containing only 20% PANI showed a significantly lower RL of 11 dB at 18 GHz [29]. Jin et al. reported a novel CNTs/Fe_3_O_4_/melamine-based carbon foam (MCF) functional material made by an in situ growth and heat treatment process of a metal-organic skeleton on a carbon-based skeleton structure. The conductivity of the CNT/Fe_3_O_4_/MCF sample reached 83.06 S.m^−1^ at the length, width, and height of 10.04 mm, 10.05 mm, and 5.00 mm, respectively. In addition, the CNT/Fe_3_O_4_/MCF also exhibited good compression cycling performance. Even after 50 compression cycles, the SE_T_ remained at 33.80 dB in the X-band, as shown in Figure 5. This is mainly due to the special structural design of the CNT/Fe_3_O_4_/MCF, which significantly improves the electromagnetic shielding performance of the functional material [30].

ICP composites not only enhance the electromagnetic shielding properties of the material, but also greatly improve other ICP properties (e.g., hydrophobicity, corrosion resistance, antimicrobial properties, etc.). In one study, Chen et al. showed that wood was turned into a good cellulose substrate by removing lignin and hemicellulose from wood. A composite material with a sandwich structure was demonstrated by the in situ polymerization of aniline on the above cellulose substrate and coating it with a polydimethylsiloxane (PDMS)/CNT layer. The material not only had excellent flame retardant properties (heat release rate (HRR) reduced by 84%, total heat release (THR) reduced by 53.4%) and significant antimicrobial activity, but also benefited from the unique layered structure and excellent conductive network, where the PDMS/CNT/PANI WA conductivity reached 18.6 S·m^−1^ and the X-band was shielded to 26 dB. In addition, PDMS enabled the material to reach a water contact angle of 105°, which greatly enhanced the durability of the material [31], and the X-band was shielded to 26 dB.

New electromagnetic shielding materials have been prepared by embedding magnetic three-dimensional carbon skeletons (3D-CS) into SiCN ceramics through plating and polymer derivatization, with excellent corrosion resistance. The distribution of the carbon conductive network and magnetic particles in the composite ceramic material becomes more uniform. In addition, the Ni distributed on the CS not only catalyzes the formation of carbon nanowires (CNWs) in the matrix, but also reacts with Si to form NiSi to affect the magnetic properties of the ceramics, resulting in a significant improvement in the EMI SE of the material. The EMI SE of the composite ceramic X-band is greater than 55 dB when the plating time is fixed at 50 min for the preparation of ceramic composites [32].

### 3.3. MXene-Based ICP Composites

MXene is a new type of two-dimensional material with a multilayer structure consisting of carbides, nitrides or carbon-nitrides. MXene has metal conductivity due to its surface hydroxyl groups or terminal oxygen. MXene-based thin film materials have shown great advantages and application development prospects in the fields of electromagnetic shielding and supercapacitors by virtue of their high electrical conductivity, ultrathin thickness, and good mechanical properties.

A multilayer structured polyvinyl alcohol (PVA)/MXene film was prepared by Jin et al. The MXene layer formed a tight conductive and thermally conductive network, which enhanced the electromagnetic shielding ability and thermal conductivity of the composite film. This multilayer film provided EMI shielding up to 44.4 dB in the X-band because the electromagnetic waves were reflected and absorbed many times within it by converting the absorbed electromagnetic waves into heat energy and then distributing the heat through excellent thermal conductivity. In addition, the laminate film was able to maintain structural stability during combustion because of its excellent flame retardancy and drip resistance. These properties make this composite film a promising material [33].

Wan et al. achieved high strength and high electromagnetic shielding of MXene-based films through a facile synthetic process. This approach utilizes the excellent mechanical strength and film-forming characteristics of the conductive polymer composite PEDOT/PSS (poly(3,4-ethylenedioxythiophene) monomer/polystyrene sulfonate). MXene nanosheets were blended with conductive PEDOT/PSS composites and the non-conductive PSS components were treated with sulfuric acid for the fabrication of nanocomposite films. These films exhibited excellent mechanical strength and were able to provide excellent EMI SE. In addition, the mechanical properties were not only unaffected by the removal of the PSS, but its EMI SE was slightly improved. With a thickness of only 6.6 μm, the MXene composite film achieved an EMI SE of more than 40.5 dB in both the X-band and ku-band [34].

In a humid environment, because of the hydrophilic nature of MXene-based materials, it may lead to a decrease in EMI SE. To solve this problem, Hao et al. fabricated superhydrophobic as well as MXene/wood (WP-MXene/delignified wood) electromagnetic shielding composites with excellent mechanical properties through a simple and efficient hot pressing and dip coating method. Because the MXene nanosheets were uniformly distributed in the layered porous structure of the wood, it had a tensile strength of about 111.4 MPa and the EMI SE was 43.4 dB in the X-band. In addition, the composite had a water contact angle of 155.1°, providing the material with excellent waterproofing properties, making it promising for a great number of applications [35].

**Figure 6:**
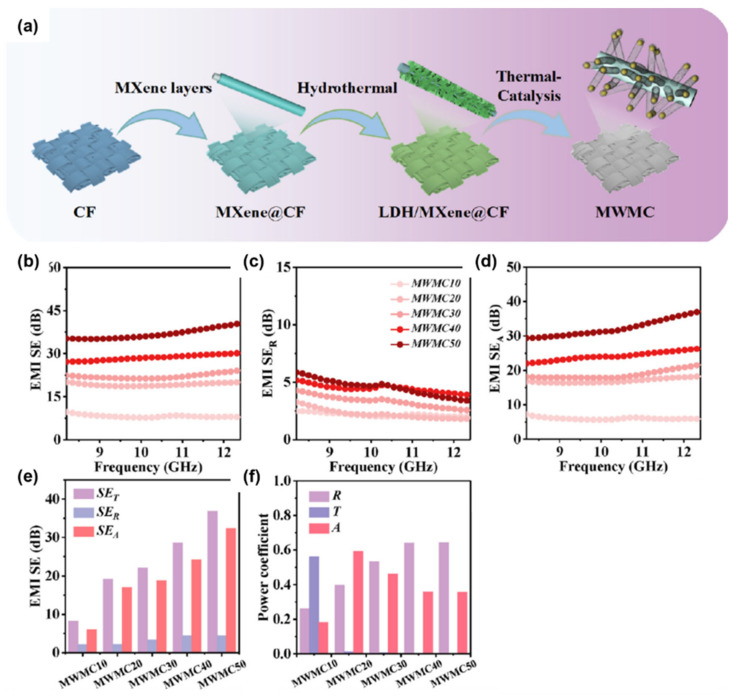
(**a**) Preparation process of the MWMC composites. The MWMC composite fillers with different proportions of (**b**) EMI SE, (**c**) EMI SE_R_, (**d**) EMI SEA, (**e**) average SE_T_, SE_R_, SE_A_, (**f**) reflection coefficient R, absorption coefficient A, and transmission coefficient T [36].

Wang et al. proposed a unique three-dimensional layered structure to fabricate MWCNTs-MXene@Cotton Fiber (MWMC) electromagnetic shielding composites. The researchers first obtained Ti_3_C_2_T_x_ MXene nanosheet suspensions by centrifugation, deionized water cleaning, and ultrasonic treatment. Then, the cotton fibers were completely immersed in the Ti_3_C_2_T_x_ MXene nanosheet suspension for five minutes by ultrasonic cleaning in acetone, ethanol, and deionized water for 30 min, and then dried at 70 °C under vacuum to obtain the MXene@Cotton Fiber composites. Finally, 1.155 g of melamine was covered with CoNi-LDH@MXene@Cotton Fiber, which was heated to 700 °C at a heating rate of 2 °C/min and maintained for 2 h under a N_2_ filled condition to obtain MWCNT-MXene@Cotton Fiber (MWMC). The MXene@Cotton Fiber (MWMC) composites and the general flow of preparation are shown in Figure 6a. The maximum SE_T_ value of MWMC50 reached 40.6 dB at a thickness of 1.0 mm, as shown in Figure 6b. In addition, PDMS was coated on the composite, which greatly enhanced the hydrophobic properties of the composite. The composite exhibited excellent electromagnetic wave absorption properties and was fully X-band absorbing [36], as shown in Figure 6.

To regulate the dipole and interfacial polarization, the construction of the conducting network, and improve the impedance matching, the growth of TiO_2_ on the Ti_3_C_2_T_x_ Mxene surface was restricted by adjusting the annealing temperature by Liu et al., and a Ti_3_C_2_T_x_/TiO_2_ heterostructured material was constructed. When the electromagnetic wave frequency was at 18 GHz and the annealing temperature was controlled at 600 °C, the EMI SE of the composite material reached 35.1 dB. In addition, TiO_2_ improved the impedance matching, which helped to reduce the electromagnetic pollution caused by secondary reflections. This work opens up a new pathway to design efficient, controllable, and green EMI shielding materials in the future [37].

Qian et al. demonstrated a new shielding composite material that produced shielding effectiveness through the interaction between Ti_3_C_2_T_x_ Mxene and tungsten-doped VO_2_ (WVO_2_). The Ti_3_C_2_T_x_/WVO_2_ composite provided a good EMI SE of 42.8 dB in the X-band (most of the electromagnetic waves were consumed by multiple internal reflections in the three-dimensional porous structure) while offering high thermal management (maximum temperature reduction from 39 °C to 32 °C). While the special porous layered structure of the Ti_3_C_2_T_x_ aerogel ensured excellent EMI shielding performance, a large amount of heat was released to ensure the viability of the phase transition. In this case, the accumulated heat of the aerogel was consumed by WVO_2_ to realize the phase transition. However, the energy converted from electromagnetic waves alone was not enough to support the phase change process. To solve this problem, a strategy to reduce the phase transition temperature by introducing tungsten atoms was proposed. When the proportion of tungsten atoms reached 1.5%, the phase transition temperature dropped to 31 °C during the endothermic process. At the same time, the process could spontaneously transfer energy between Ti_3_C_2_T_x_ and WVO_2_ without external stimulation. In addition, it is noteworthy that part of the conductive pathway consisting of Ti_3_C_2_T_x_ Mxene was blocked to some extent with the introduction of WVO_2_, and therefore, the shielding properties of the Ti_3_C_2_T_x_ aerogel were weakened. However, with the change in temperature from 300 K to 308 K, the conductivity of WVO_2_ increased sharply after the phase change, at which time a good conductive channel was reconstructed by WVO_2_, and thus the EMI shielding performance of the composite was greatly improved. This not only solved the problem of thermal accumulation, but also improved the EMI shielding performance [38].

Qu et al. constructed a flexible AgNF/MXene/AgNW (AMA) electromagnetic shielding material by using nanosilver flakes (AgNFs) as the reflective layer, Ti_3_C_2_T_x_ MXene as the loss layer, and AgNWs as the thermal conductivity and shielding layer. In the X-band, the composite exhibited an EMI SE of 70.96 dB even at an extremely thin thickness (25 g/m^2^) and was 3.5 times higher than the general standard (20 dB). In addition, the AMA composite could withstand high temperatures from 25 °C to 100 °C, and the AMA composite could reach a tensile strength of 44.4 MPa and a Young’s modulus of 0.6 GPa [39]. The electromagnetic shielding properties of various ICP composites are listed in Table 2.

## 4. Conductive Polymer-Based Composites (CPC)

CPCs are materials that combine a polymer matrix with a conductive filler such as CNTs, graphene, or metal particles. CPCs exhibit a unique combination of properties such as high electrical conductivity, good mechanical properties, and light weight, making them suitable for a variety of applications. One of the main advantages of CPCs is their high electrical conductivity, which can be tailored to various applications. The conductive fillers in the polymer matrix form a continuous network that allows for efficient electron transfer. This makes CPCs well-suited for applications requiring conductive properties such as antistatic coatings and sensors. CPCs also exhibit good mechanical properties including high strength and stiffness, which makes them suitable for structural applications. Adding conductive fillers to a polymer matrix allows it to withstand greater loads and stresses. In addition, the lightweight properties of CPCs make them well-suited for applications that require weight reduction such as the aerospace and automotive industries [40].

### 4.1. Factors Affecting the Performance of CPC for EMI Shielding

#### 4.1.1. Percolation Threshold

**Figure 7:**
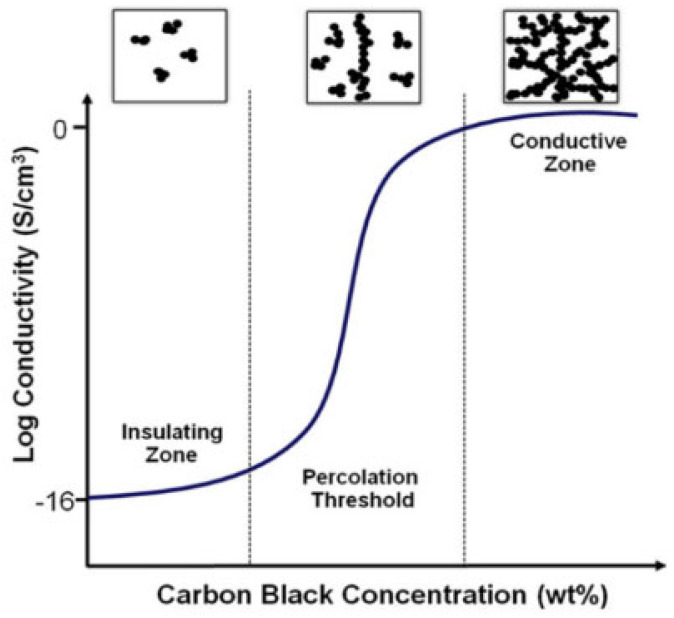
Classical percolation curve [41].

Adding conductive materials as fillers can significantly increase the dielectric constant of the insulator matrix. The formation of interconnected conductive networks within the CPC can be explained by the electrical leakage threshold theory. When the amount of conductive filler in an insulating polymer reaches a critical concentration, a network of conductive filler forms in the polymer matrix, thus transforming the insulating polymer into a conductive polymer composite. This phenomenon is known as the “percolation transition” and the minimum concentration of conductive filler required to achieve this transition is known as the “percolation threshold”. However, as the filler concentration increases, the conductivity of the composite decreases as the filler concentration exceeds the “percolation threshold” [41,42], as shown in Figure 7.

The percolation threshold σ is calculated as in Equation (13)

(13) $$\begin{array}{l} \sigma =\sigma_{1}{\left(\varphi_{c}-\varphi \right)}^{t},\varphi <\varphi_{c}\\ \sigma =\sigma_{2}{\left(\varphi -\varphi_{c}\right)}^{-s},\varphi >\varphi_{c} \end{array}$$

where $\sigma_{1}$ is the filler conductivity, $\sigma_{2}$ is the insulating polymer matrix conductivity, φ is the filler concentration, and $\varphi_{c}$ is the percolation transition filler concentration. t, s are parameters elaborating the number of inter-filler connections and conductive network arrangement under percolation threshold (i.e., particle dispersion and 2D or 3D systems). Typically, for a 2D network, t = 1.1–1.3 and s = 1.1–1.3; for a 3D network, t = 1.6–2.0 and s = 0.7–1.0, but the experimental t was 1–12. The cause of this deviation is yet to be fully understood, but the most plausible explanation is that when “t” is greater than 2, it indicates the formation of a 3D network, whereas a “t” value less than 2 suggests the formation of a 2D network [43,44].

#### 4.1.2. Conductive Packing Concentration

For CPCs with a conductive filler content below $\varphi_{c}$, the dielectric constant and dielectric loss coefficient are minimally affected in the measured frequency range, but increase with increasing conductive filler content. The percolation threshold ($\varphi_{c}$) is highly dependent on the geometric characteristics of the conductive fillers including their size, shape, orientation, and conductivity. In the case of conductive polymer composites with randomly dispersed fillers, an increase in the filler aspect ratio leads to a decrease in the $\varphi_{c}$ value. In insulating polymer networks composed of conductive nanofillers, the electron transport is accomplished between the conductive fillers by hopping or tunneling effects [45,46,47,48,49]. At the same time, the reflection or absorption mechanism of CPC is related to the properties of the filler.

It has been shown that increasing the concentration of vapor grown carbon fiber (VGCNF) in a polyvinylidene difluoride (PVdF) matrix can improve the absorption of electromagnetic waves in the composites because at higher filler concentrations, the voids between the fillers will be reduced and the absorption of electromagnetic waves will be further enhanced and the absorption capacity of the composite material for electromagnetic waves will be further enhanced [50,51]. Arjmand et al. conducted a comparative study of the EMI SE of polystyrene (PS)/MWCNT composites fabricated by compression molding and injection molding techniques. Their results showed that the greatly enhanced connectivity between MWCNT fillers in the PS/MWCNT composites by the compression molding technique led to increased polarization in the thinner PS matrix region around the MWCNTs, resulting in composites exhibiting higher actual dielectric constant values and superior EMI SE. This further improved the EMI shielding absorption capability of the compression molded composites. In contrast, the composites produced by the injection molding technique showed poor performance in this respect [52,53]. In injection molded composites, when the arrangement of MWCNTs increases, the connectivity between them decreases. The change in connectivity is closely related to the absorption of electromagnetic waves by the composite material. Specifically, the connectivity of the filler in the matrix affects the absorption of electromagnetic waves, while the concentration of conductive fillers affects the reflection of electromagnetic waves.

#### 4.1.3. Distribution and Selective Positioning of Conductive Fillers

The distribution of the filled conductive material in the polymer matrix is influenced by both the concentration of the filler and the interaction of the filler with the polymer. At low concentrations, the filler–polymer interaction is negligible, but higher filler concentrations lead to significant changes in filler distribution, polarity matching, and polymer–filler interactions, resulting in a conductive network structure. However, if the filler concentration is too high, the fillers can clump together due to van der Waals interactions, adversely affecting the physical properties and material cost. Therefore, these two factors need to be balanced when filling conductive materials [54,55,56].

By selectively positioning conductive fillers in the polymer system, $\varphi_{c}$ can be reduced. Preferential positioning of conductive fillers can increase their local concentration, resulting in an effective conductive network [57]. Selective positioning is mainly achieved by two strategies, a separated structure and double permeable structure, which in turn reduce the packing concentration. Yan et al. achieved an EMI SE of 28.3–32.4 dB and exhibited excellent conductivity of 34 S·m^−1^ in a thermally reduced graphene oxide (TGRO)/ultrahigh molecular weight polyethylene (UHMWPE) composite with a TGRO content of only 0.660 vol.% using the separate structure approach [58]. The reduced graphene oxide (RGO)/PS composite (2.5 mm) with a polycrystalline facet separation structure and conductive filler content of 3.47 vol.% exhibited an excellent EMI SE of 45.1 dB in the X-band and a conductivity of 43.5 S·m^−1^, mainly because the polycrystalline facet separation structure provided more interfaces [59]. In the double-permeable structure, the conductive filler was added to the continuous component of two incompatible polymer blends, also forming a complex conductive structure and thus enhancing the shielding effectiveness [60,61]. PC/ polystyrene-acrylonitrile (SAN) 60/40 MWCNT double-permeable CPCs prepared by the melt-compounding method were used by Bizhani et al. The EMI SE in the X-band of a 10 mm thick sample reached 25–29 dB and the electrical conductivity was 0.083 S·cm^−1^ at a MWCNT content of 1 wt% [62].

Despite their superiority in many aspects, composites with isolated and double-permeable structures still have some disadvantages. In isolated CPCs, the diffusion of molecules in the polymer matrix is greatly inhibited due to the obstruction of the conductive layer, which makes the adhesion of the polymer weaker. In the dual-permeable structure, poor interfacial interaction of CPC is caused by the incompatible interfaces between different blends. Therefore, the above problems can be improved by changing the processing/manufacturing conditions. Wu et al. showed that CNT/PP (polypropylene, polypropylene) produced using the bias method at 180 °C and 100 MPa under high temperature and pressure conditions could effectively suppress the interfacial defects and improve the connectivity of CNTs [63]. The SE_T_ of the biased CNT/PP composite prepared by solid-phase molding was 17.1 dB when the CNT content was 0.3 wt%, while a similar SE_T_ was only achieved when the CNT content was 5 wt% in CNT/PP composites prepared by the melt blending method. The expected fillers could easily aggregate on a well-defined interface produced by mixing polymers of different polarity [64].

#### 4.1.4. Shape and Chemical Properties of Conductive Fillers

However, the conductive network of CPC composites causes the motion of polymer molecular chains to be inhibited. Therefore, the addition of a conductive filler will reduce the toughness and ductility of the CPC. Therefore, some studies have attempted to add hybrid fillers with better conductivity to reduce the percolation threshold of CPC and thus improve the mechanical properties. In addition, the physical morphology and the dispersion of the conductive filler in the matrix also have an effect on $\varphi_{c}$ [65]. Joseph et al. showed that silicone rubber (SR) filled with 2 wt% of CNT and CNF and CNF wafers, respectively, where the CNF wafer-filled SR exhibited the best EMI SE when the electromagnetic wave frequency was 2.0–18.4 GHz, and when the thickness of the CNF wafer increased by 0.02 mm (0.73 mm–0.75 mm), and that the EMI SE of the composite material increased by 3.87 dB (35.08 dB–38.95 dB) at this time. In addition, the CNF filled SR had a better EMI SE than the CNT filled SR. This is related to the wavelength of the electromagnetic wave, because in the measured frequency region, the wavelength of the electromagnetic wave is almost equal to the length of a CNF, but longer than a CNT [66].

By altering the chemical proximity and functionalization of the carbonaceous fillers, their interaction with the polymer matrix and their dispersion can be improved, thus affecting their $\varphi_{c}$, impedance matching, and EMI shielding properties in the composite [67,68,69,70,71,72]. Graphene foam (GF) was functionalized using dodecyl benzene sulfonic acid (DBSA) with the aim of improving its wettability and bonding with PEDOT:PSS (3,4-ethylenedioxythiophene monomer polymer: polystyrene sulfonate). The PEDOT:PSS/GF composites prepared using this method exhibited negative dielectric constants due to the presence of polarized charge leaving domains at the interface. The optimized PEDOT:PSS/GF composite was enhanced by the addition of DBSA, and the composite finally exhibited an excellent EMI SE of 91.9 dB [73,74]. Liang et al. showed that with the addition of functionalized graphene to the epoxy resin matrix, a lower $\varphi_{c}$ could be obtained when the graphene content was 0.52 vol.%, and an electromagnetic wave shielding effect of 21 dB could be achieved at 8.8 vol.% in the X-band [75].

### 4.2. Common CPC Materials

**Figure 8:**
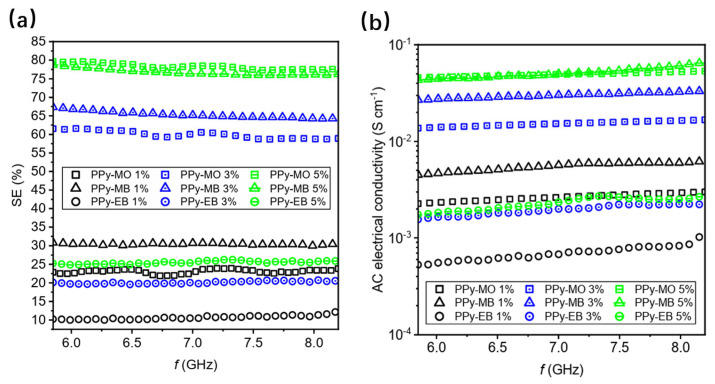
Cured at 25 °C. (**a**) EMI shielding efficiency of PPy nanostructures in the C-band range; (**b**) AC electrical conductivity [76].

PPy nanostructures have excellent electromagnetic shielding properties. Three different one-dimensional nanostructures (nanotubes, nanoribbons and nanofibers) of polypyrrole were compared by Dušan Kopecký et al. and were synthesized in the presence of three different dyes (methyl orange, methylene blue, and chromium black T). These nanostructures have high electrical conductivity, high thermal stability, and high aging resistance and can be used to prepare lightweight and flexible composites. Measurements in the paper revealed that polypyrrole nanotubes and nanoribbons could shield nearly 80% of incident radiation at 5% (*w*/*w*) in the C-band (5.85–8.2 GHz) at 25 °C. The conductivity also reached approximately 10^−1^ s.cm^−1^. The shielding efficiency of polypyrrole nanofibers, on the other hand, was lower and mainly depended on reflection [76], as shown in Figure 8. In another of their studies, the authors found that the morphology and conductivity of PPy had a significant effect on the shielding efficiency. They studied three different shapes of PPy, namely spherical, nanotube, and microbarrel, and found that the nanotube shape had the highest conductivity (60.8 S·cm^−1^) and shielding effectiveness, while the PPy microbarrel had the highest absorptive capacity. The deprotonation of PPy reduced its conductivity and shielding efficiency, and the concentration of PPy and the curing temperature of the substrate also had an effect on the shielding efficiency [77].

**Figure 9:**
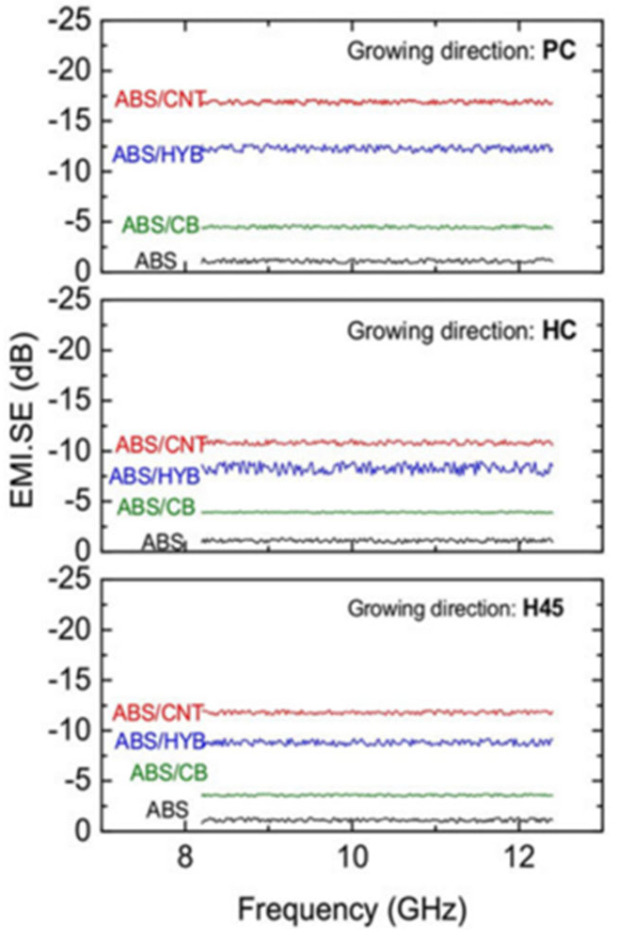
EMI SE of the ABS/carbon composites versus growth direction: vertical (**top panel**), horizontal concentric (**middle panel**), and horizontal alternating (**bottom panel**) [80].

Schmitz et al. produced a CPC based on acrylonitrile-butadiene-styrene (ABS) using a mixture of carbon black (CB) and MWCNTs as the conductive filler and compared its EMI SE with MWCNTs or CB when used as the conductive filler, respectively. The blend of MWCNTs and CB allowed the composite to exhibit a good EMI SE even at a lower MWCNT content. The ABS composite containing 3 wt% MWCNT:CB (50:50) exhibited good melt flow index (12.10 g/10 min) and EMI SE (23.8 dB). When the mixing ratio was 75:25, the ABS composite achieved an EMI SE of 29.4 dB [78,79]. In addition, the team prepared three composites, ABS/CNTs, ABS/CB, and ABS/CNT/CB, using a fused deposition model. The EMI SE of the ABS composites at a filler loading of 3 wt% followed the order of pure ABS < ABS/CB < ABS/HYB < ABS/CNT, independent of the growth direction. Figure 9 shows the effect of the ABS CPC growth direction on its EMI SE. It can be seen that the samples prepared along the PC direction showed stronger attenuation when electromagnetic waves were incident on each surface of the ABS/carbon composites because they had the highest bulk conductivity [80].

**Figure 10:**
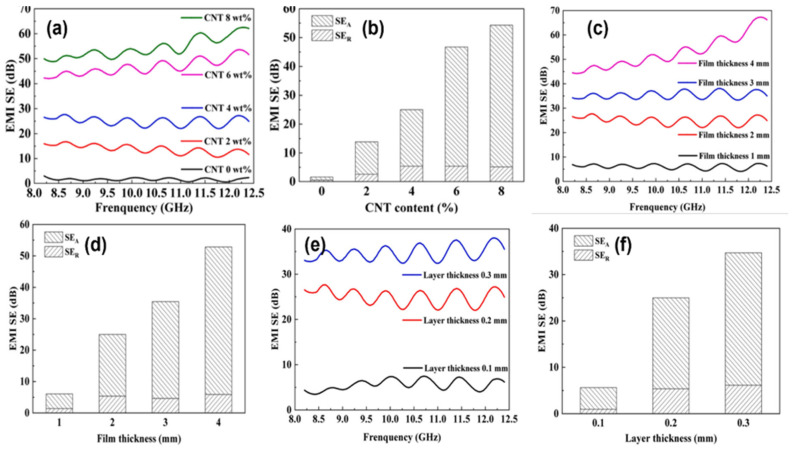
(**a**) Effect of the CNT content on the EMI SE of CNT/PLA films, (**b**) different SE_A_ and SE_R_ of CNT/PLA films with different CNT contents. (**c**) Effect of film thickness on the EMI SE of CNT/PLA films, (**d**) different SE_A_ and SE_R_ of CNT/PLA films with different film thicknesses. (**e**) effect of layer thickness on the EMI SE of CNT/PLA films when printed by the FDM technique, (**f**) different SE_A_ and SE_R_ of CNT/PLA films when printed by the FDM technique with different layer thicknesses [81].

Given that the fused deposition model (FDM) has the advantages of low cost of use, ease of operation, and wide applicability, Yang et al. proposed the use of the FDM process to fabricate a multifunctional CNT/polylactic acid (PLA) film with excellent EMI SE along with excellent electrothermal properties. The researchers investigated the effects of parameters such as CNT content and the thickness of the FDM-printed film on the EMI SE of the composite. With the increase in CNT content, the conductive network in the CNT/PLA FDM-printed films was improved to enhance the EMI SE of the composites. When the CNT/PLA film thickness was 4 mm and the CNT content was only 4 wt%, the EMI SE of the composites reached 68 dB at a frequency of 12.3 GHz, as shown in Figure 10c. Although the film thickness was the same, the different layer thicknesses when printed by FDM technology still affected the final EMI SE of the composite, as shown in Figure 10e. In addition, the composite had good electrical heating properties. The heating temperatures of the composites with CNT contents of 4 wt% and 8 wt% were 67.8 °C and 139 °C, respectively, when 16 V was applied to the CNT/PLA film. This study greatly expands the potential of the development and application of electromagnetic shielding materials and FDM technology [81].

Cai et al. prepared a multifunctional elastic foam with the excellent electrical conductivity and good mechanical properties of CNTs. The PDMS/EM/CNT composite foam was first dried by stirring the PDMS/CNT mixture at high temperature, and then the curing agent and expanded microspheres (EM) were added and pressed at 110 °C and 4 MPa for 30 min to obtain the PDMS/EM/CNT composite foam material. The mechanical properties and electrical conductivity of the composite were significantly improved, and it had an excellent EMI SE along with high stability, mainly due to the addition of EM. However, the thermal expansion microsphere content needs to be controlled because the foam cannot be obtained at a low thermal expansion microsphere content, while the composite material is difficult to cure completely at high thermal expansion microsphere content. The EMI SE of the PDMS/CNT/EM material reached 43 dB when the EM and CNT contents were 50 vol.% and 1.74 vol.%, respectively [15].

**Figure 11:**
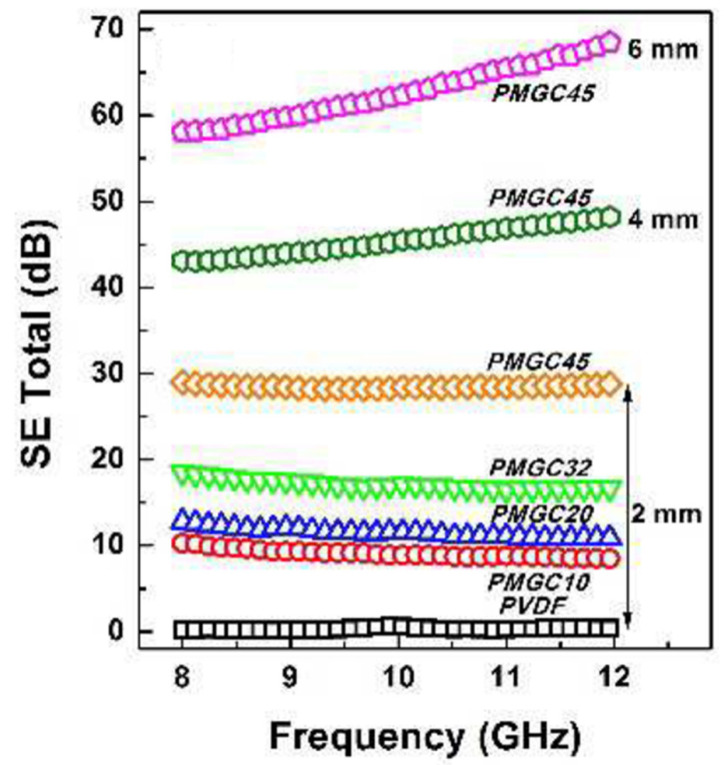
SE_T_ with different contents of carbon filler and different sample thicknesses [82].

Ma et al. reported a self-cleaning EMI shielding material, namely a porous superhydrophobic PVdF/MWCNT/GN composite (PMGC). The material had a carbon filler content of 4.5 vol.% (MWCNT:GN = 1:1), a thickness of 2 mm, and a multiscale rough-like morphology on the material surface, exhibiting a water contact angle of 155.4 ± 2.7 and an EMI SE of 28.5 dB [82], as shown in Figure 11.

Zhou et al. reported the successful preparation of water polyurethanes (WPUs) containing MWCNTs and Ni-coated multi-walled MWCNTs (Ni@MWCNTs) by emulsion mixing and drop coating. Ni@MWCNTs/MWCNTs are commonly used as conductive and magnetic fillers because of their excellent properties such as large aspect ratio, high electrical conductivity, and high permeability. The dispersion of the conductive filler is greatly enhanced by the nonionic surfactant in the MWCNT dispersion, so Ni@MWCNTs/MWCNTs constitute a nearly perfect conductive, electromagnetic network in WPUs. As a result, the EMI SE of the composite reached an astounding 77.2 dB [83].

Chang et al. developed a flexible and porous Cu/Poly (L-lactic acid) (PLLA) fiber membrane with a thickness of only 15 μm, and the Cu/APLLA composite could be obtained after acetone treatment. The mechanical strength of the Cu/APLLA composite membrane was greatly enhanced compared to Cu/PLLA. The material had excellent permeability and electrical conductivity of 9472 S·cm^−1^. Meanwhile, due to the special porous structure of the polymer substrate, it was more conducive to the diffusion of conductive materials, so its absolute shielding effectiveness reached 7798 dB cm^2^·g^−1^ in the H-band and 8073 dB cm^2^·g^−1^ in the Ku-band [84].

Liu et al. used a mixture of isomeric polypropylene (iPP) and polyethylene-1-octene (POE) to form an segregated CPC and successfully dispersed MWCNTs in the continuous iPP phase using a melt-bonding technique to form an excellent conductive network where the $\varphi_{c}$ of the MWCNTs was as low as 0.24%. At 3.0 vol.% MWCNTs and a 1.2 mm thickness, the composite exhibited an EMI SE of 25 dB [85]. Zha et al. investigated the selective distribution limitation of MWCNTs at the interface of a PVDF and ethylene-α-octene block copolymer (OBC). They found that the $\varphi_{c}$ of the PVDF/OBC/MWCNT nanocomposites was reduced by 23% compared to the PVDF/MWCNT nanocomposites. The ${\varepsilon^{\prime }}_{r}$ values of the PVDF/OBC/MWCNT composite system increased with the increase in MWCNT dosage, but the tanδ values were lower than the PVDF/MWCNT composites. The EMI SE of PVDF/OBC/MWCNT reached 34 dB at 2.7 vol.% MWCNTs. Because of the accumulation of charge carriers, a strong interfacial polarization occurred, resulting in a significant enhancement in the dielectric properties and EMI SE of the material [86].

Jae Ryung Choi et al. demonstrated two GN composites with a thickness of only 43 μm, GN/PVA/cellulose and GN/polyacrylic acid (PAA)/cellulose, both of which were fabricated on a cellulose substrate by the spray deposition technique. The addition of PVA and PAA increased the adhesion of GN to the cellulose substrate and drove the graphene to form an effective conductive network on the cellulose base, resulting in a composite with an extremely low resistivity of 0.003 Ω·m, an EMI SE of 28.3 dB for GAC at 10 GHz, 17.4 dB for SE_A_, and 10.9 dB for SE_T_. This study has good potential for application in wearable smart electronic devices [87].

**Figure 12:**
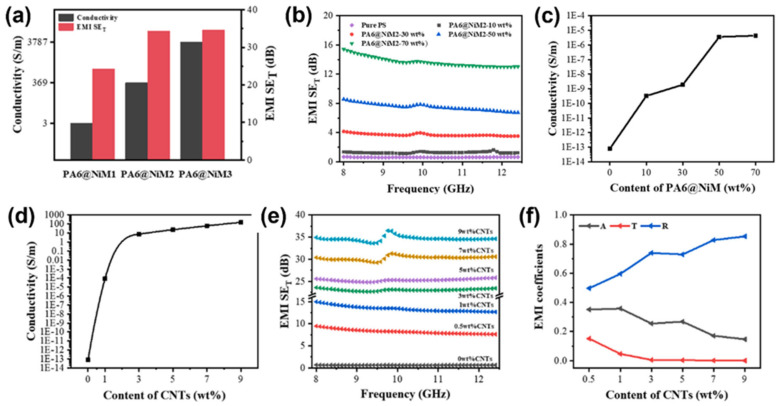
(**a**) Conductivity and EMI SE of PA6 @NiM1 (23.3 wt% Ni), PA6 @NiM2 (39.2 wt% Ni), and PA6@NiM3 (46.4 wt% Ni). (**b**) Effect of different PA6 @NiM contents on the EMI SE of PS/PA6 @NiM. (**c**) Effect of different PA6 @NiM contents on the electrical conductivity of PS/PA6 @NiM. (**d**) Effect of different MWCNT contents on the conductivity of PS/MWCNT. (**e**) Effect of different MWCNT contents on the EMI SE of PS/MWCNT. (**f**) R, T, and A of PS/MWCNT in the X-band range with different MWCNT contents [88].

In a study by Wu et al. a composite PA6 @NiM/MWCNT/PS with excellent electromagnetic shielding ability was prepared. This was obtained by depositing (Ni) particles on the surface of nylon microspheres (PA6) and then synthesizing with PS containing MWCNTs. When the thickness of the surface Ni coating of PA6 @NiM was 0.44 μm and the Ni content was 39.2 wt%, the conductivity of PA6 @NiM = 369 S·m^−1^, as shown in Figure 12a. As shown in Figure 12b, PA6 @NiM can optimize the conductive path of MWCNT and enhance the material conductivity, thus enhancing the EMI SE of the composite. When the contents of PA6 @NiM and MWCNT were 10 wt% and 7 wt%, respectively, the EMI SE of PA6 @NiM/MWCNT/PS reached 46.9 dB, which was 12.7 dB higher than that of pure PA6 @NiM_2_, as shown in Figure 12a, and was 16.64 dB higher than 7 wt% MWCNT/PS, as shown in Figure 12c [88]. The electromagnetic shielding properties of various CPC composites are listed in Table 3.

### 4.3. Conductive Anisotropy CPC

Conductive fibers show anisotropy in the polymer matrix, and the arrangement of conductive fibers is usually divided into two types: longitudinal arrangement and transverse arrangement. Longitudinal alignment is the arrangement of the conductive fibers in the polymer matrix perpendicular to the surface of the composite material, that is, the length of the conductive fibers in the composite material is perpendicular to the surface. Transverse alignment guides the electrical fibers in the polymer matrix in an arrangement parallel to the surface of the composite, that is, the length of the conductive fibers in the composite is parallel to the surface. When the conductive fibers are aligned along the length direction in the composite, the CPC material has better conductivity in the length direction and poorer conductivity in the width and thickness directions. Conversely, when the conductive fibers are aligned perpendicular to the surface in the composite, the CPC material has better conductivity in the thickness direction and poorer conductivity in the length and width directions. This anisotropic performance allows CPC materials to be used for electromagnetic shielding in different directions. For example, when electromagnetic waves are irradiated from above, the thickness direction of the CPC material can play a shielding role, while when electromagnetic waves are irradiated from the side, the length and width direction of the CPC material can play a shielding role [89].

**Figure 13:**
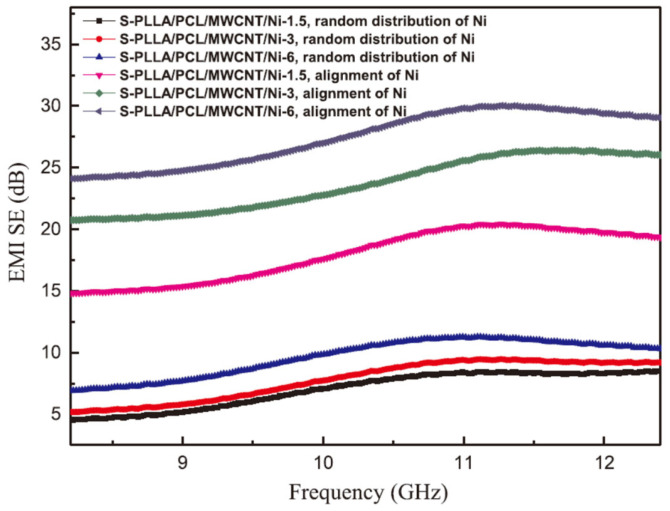
Effect of the Ni particle arrangement on the EMI SE values of PLLA/PCL/MWCNT/Ni CPC [90].

Shi et al. achieved conductive anisotropy in an S-PLLA/PCL/MWCNT/Ni CPC by applying a magnetic field of 47.5 mT at 100 °C for 30 min [90]. After successfully achieving anisotropy in the conductive material, the researchers observed that higher σ values were observed in the direction of alignment parallel to the Ni-particle chains, improving the EMI SE values. Composites with an ordered arrangement of nickel chains exhibit better EMI shielding performance with EMI SE values in the range of 19–24 dB compared to composites with isolated structures with randomly distributed Ni. Because the formation of nickel particle chains improves the electrical conductivity of the material, this in turn improves the absorption and reflection of electromagnetic waves by the nanocomposite, greatly enhancing its EMI SE, as shown in Figure 13.

A DC electric field causes the single-walled carbon nanotubes (SWCNTs) to be ordered in the epoxy/SWCNT composites. Compared with the randomly arranged SWCNT composites, this composite with an ordered SWCNT arrangement has higher electrical conductivity. Under the action of a DC electric field, the SWCNTs showed a stronger tendency of parallel arrangement. Thus, the conductivity of the composite in the parallel direction was 4.6 × 10^−6^ S·m^−1^, much higher than that of 9.3 × 10^−9^ S·m^−1^ in the vertical direction at a SWCNT content of 0.5 wt% [91].

## 5. Lightweight Polymer Composites and Multi-Component Systems

### 5.1. Lightweight Polymer Composites

Conductive filled polymer foam is widely used in the field of electromagnetic shielding due to its good mechanical and physical properties. Compared to rigid materials, flexible materials perform better in terms of recovery and leakage thresholds. The physical foaming method, chemical foaming method, and freeze-drying method are the three most important methods for preparing foam composites. The physical foaming method is usually used to prepare foam composites by injecting foaming agents (critical carbon dioxide, expanding microspheres, etc.) directly into the substrate [92,93]. The principle of chemical foaming is the bubble produced by the chemical reaction of the substance, which results in good electromagnetic shielding performance. Freeze-drying is an emerging advanced manufacturing technology because of its ability to fine-tune macroscopic material structures and because it has become an important part of the future development of new materials [94,95].

A polyurethane foam nanocomposite containing Ketjen carbon black (K-CB) was prepared by the dip-coating method. To avoid the cohesion of K-CB during the infiltration of polyurethane foam, the researchers used polyethylene glycol (PEG 4000) as an ionic surfactant. The results showed that the surfactant had a negligible effect on the polyurethane foam nanocomposites. The polyurethane (PU) foam composites exhibited 65.6 dB of EMI SE performance at a K-CB loading of 2 wt% [96].

**Figure 14:**
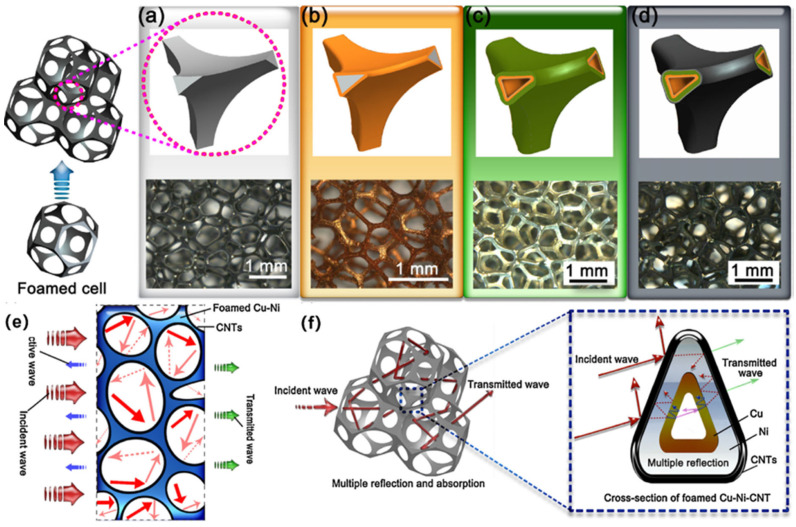
The manufacturing process of this foam composite (**a**–**d**). Schematic representation of microwave transmission on the foam Cu-Ni-CNT composite (**e**). The influence of the special structure inside the foam material on EMI (**f**) [103].

Foam metal is an emerging material with a three-dimensional network structure consisting of an interconnected metallic skeleton and an internal porous structure. Foam metal not only has excellent electrical conductivity and magnetic properties, but can also make electromagnetic waves in the internal multiple reflections and scattering because of its special porous structure, so the foam metal material has a high EMI SE. Compared with the traditional 2D metal network, foam metal has higher electromagnetic wave shielding effectiveness [97,98,99,100]. Foam metal has the characteristics of lightweight, high strength, and good electromagnetic shielding. Thus, it can be used to make heat dissipation structures for precision instruments. Research shows that titanium foam with a porosity of 86–90% has an obvious electromagnetic shielding effect and performs well in low frequency [101]. The electromagnetic shielding performance of titanium foam is mainly characterized by reflection loss in the low-frequency region and absorption loss in the high-frequency region. To prepare lightweight multilayer EMI shielding materials, researchers have chemically coated copper, electroplated nickel, and electrophoretically deposited carbon nanotubes on the surface of open-cell polyurethane foam. The experimental results showed that the shielding effect increases when the pore density and pore diameter of the composite increases, as shown in Figure 14 [102]. Yan et al. prepared RGO/Cu foam composites by electroless plating and electrodeposition, and the mechanical strength and EMI SE of the RGO/Cu foam were greatly improved compared to that of pure copper foam. With an RGO content of only 0.096 wt%, where RGO was uniformly dispersed in the copper foam matrix and formed only nano-sized cuprous oxide particles on the functional surface of RGO, the composite achieved an EMI SE of 35 dB at the X-band (the main shielding mechanism was absorption loss) [103].

**Figure 15:**
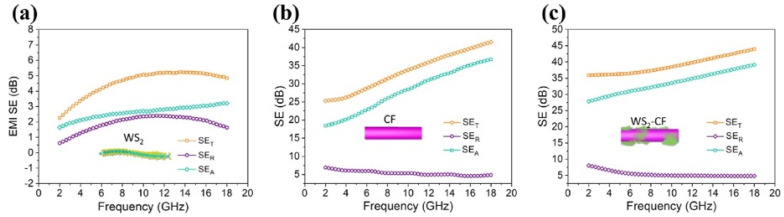
(**a**) EMI SE for pure WS2 (3 mm). (**b**) EMI SE for pure CF (3 mm). (**c**) EMI SE for WS2-CF (3 mm) [104].

Zhang et al. synthesized a WS_2_-carbon fiber (WS_2_-CF) electromagnetic shielding composite by implanting WS_2_ with a multiphase structure on the surface of carbon fiber (CF). When the thickness of the composite was 3.00 mm, the EMI SE of the WS_2_-CF composite could reach 36.0 dB at 2 GHz, which is much higher than the 25.5 dB of pure CF, as shown in Figure 15. This study provides a new idea for flexible and wearable shielding materials. The EMI SE of the WS_2_-CF composite will be almost unaffected after being subjected to wear and mechanical deformation, so the composite has a long service life [104].

The researchers prepared lightweight (flexible methyl vinyl silicone rubber, VMQ)/MWCNTs/Fe_3_O_4_ nanocomposite foams by a supercritical carbon dioxide (Sc-CO_2_) foaming process [105]. After the introduction of Fe_3_O_4_ nanoparticles, the main shielding mechanism of the flexible silicone rubber foam changed to absorption loss. The foam material maintained excellent EMI SE stability even when it was repeatedly bent. In addition, the EMI SE absorption capacity of the VMQ/MWCNT/Fe_3_O_4_ nanocomposite foam ass significantly improved. This is due to the special structure of the foam, which plays a role in EMI SE loss, and the good magnetic properties of Fe_3_O_4_ nanoparticles. In another study, the researchers proposed constructing graphene particles/reduced graphene oxide foam/epoxy resin (GNPs/rGO/EP) composite electromagnetic shielding materials with 3D porous structure. The three-dimensional rGO foam embedded in GNP constructed a 3D network structure with good electrical and thermal conductivity in EP [106]. In the above nanocomposites, the rGO network acts as a substrate for GNPs, which greatly facilitates the formation of conductive networks. The GNPs/rGO/EP nanocomposites have a unique network structure, resulting in an EMI SE of 51 dB at the X-band compared to rGO/EP and GNP/EP by 292% and 240%, respectively.

In another study, a lightweight and durable composite material consisting of CNT and polyimide (PI) foam was proposed, which not only had good EMI SE but also good heat resistance. First, CNT was introduced into water-soluble PAA and the CNT/PI foam was prepared by in situ polymerization. Then, to effectively and uniformly disperse the CNT to form a conductive network, the researchers used polyvinylpyrrolidone (PVP) as a surfactant, which greatly enhanced the material’s conductivity. The prepared composite foam had low density, high stability, excellent mechanical properties, and an EMI SE of 41.1 dB [107]. Jia et al. used PU film containing calcium alginate (CA) and AgNWs as a novel electromagnetic shielding film. Since PU and CA are transparent to electromagnetic waves, the electromagnetic shielding function was provided by the AgNWs. The CA/AgNW/PU film exhibited an excellent EMI SE of 31.3 dB in the frequency range of 4–18 GHz, with an area density of 174 mg·m^−2^ for AgNW and a film transmittance of 81% [108]. Oh et al. investigated the EMI SE of polyethylene terephthalate (PET) films after coating a layer of silver nanoparticles (Ag-NP) on the surface in the frequency range of 0.1~1 GHz. The EMI SE of the AgNP/PET composite was 60.5 dB at 0.1 GHz and 54.7 dB at 1.0 GHz [109]. Guo et al. fabricated a composite foam with a sandwich structure using electroless plating and compression molding. First, an aramid-carbon hybrid fabric covered with a Co-Ni coating was used as the surface layer of the sandwich structure, and then a porous polyurethane (PU) foam containing different contents of CNTs was used as the core layer. When the content of CNTs was only 3 wt%, the CNTs built an excellent conductive network in the middle layer. With the synergistic effect of reflection from the surface layer and absorption from the middle layer, the aramid-carbon hybrid fabric/CNT@PU composite exhibited an excellent EMI SE of 73.9 dB at the X-band. In addition, the aramid-carbon hybrid fabric/CNT@PU composite achieved a tensile strength of 13.82 GPa and exhibited excellent thermal insulation properties from 0 to 150 °C with a very low thermal conductivity of 0.069 W·(m·K)^−1^ [110].

Because of the excellent electrical conductivity, hydrophilicity, and flexibility of MXene composite foams, the application areas of 3D MXene-based foams have been greatly expanded such as sensors, photothermal conversion, and other fields.

An absorption-based EMI shielding material was reported by Xu et al. The Ti_2_CT_x_ MXene/PVA porous composite foam was prepared by the freeze-drying method using porous Ti_2_CT_x_ (f-Ti_2_CT_x_) MXene and polyvinyl alcohol as the raw materials. The specific shielding efficiency was found to be 5136 dB cm^2^·g^−1^ with an ultra-low filler volume. This material has good impedance matching due to its special porous structure and f-Ti_2_CT_x_ layer structure, is not only lightweight and has a higher material strength, but has excellent EMI SE. When the thickness of the material was only 5 mm, the electromagnetic shielding effectiveness reached 26~33 dB [111]. Wu et al. developed a high-performance foam using MXene, a promising material that exhibited excellent compressibility, durability, and an electromagnetic shielding effectiveness of up to 53.9 dB. They constructed a three-dimensional MXene aerogel structure by freeze-drying and with the help of sodium alginate (SA). The researchers coated the aerogel with a layer of PDMS, which was used to maintain and improve the stability of the porous structure. The combination of MXene’s high electrical conductivity and its laminar structure formed an excellent conductive network. In addition, the conductive network was further strengthened by the addition of PDMS [112].

### 5.2. Multicomponent Systems

A multi-component electromagnetic shielding system is a system that combines multiple materials to create a shield that provides superior protection from electromagnetic radiation. In a multi-component electromagnetic shielding system, a variety of components can be used including conductive, magnetic, and dielectric materials [83,113,114]. Materials with high conductive properties can effectively reflect or absorb electromagnetic radiation. Magnetic materials are capable of redirecting or absorbing electromagnetic radiation through magnetic induction. Dielectric materials have high dielectric constants and are capable of absorbing and dissipating electromagnetic radiation. In multi-component systems, these materials are combined to create a shield that is more effective than any single component. For example, a shielding system may use a conductive layer on the surface, a magnetic layer underneath the conductive layer, and a dielectric layer underneath the magnetic layer. Multi-component electromagnetic shielding systems can be customized to meet the needs of specific applications. By carefully selecting and combining materials, a shield can be created that provides excellent protection against electromagnetic radiation [115].

Xu et al. prepared a heterogeneous asymmetric bilayer composite (HADC) by chemical plating and stirred foaming, in which the absorber layer consisted of Fe_3_O_4_@rGO/WPU, while Ni-Co-P@FC (Ni-Co-P coated filter cotton (FC))/PANI@PDMS served as the reflective layer. The Fe_3_O_4_@rGO/WPU foam in the top layer exhibited excellent impedance matching and EMI SE using the porous internal structure, while the dense conductive network formed by the Ni-Co-P in the bottom layer ensured that the material had excellent electrical conductivity. The EMI SE of this HADC was 36.6 dB as well as a very high absorption coefficient of 0.94. The preparation of this material is not only low cost but also environmentally friendly, broadening the application of porous structures in the field of electromagnetic shielding [116].

**Figure 16:**
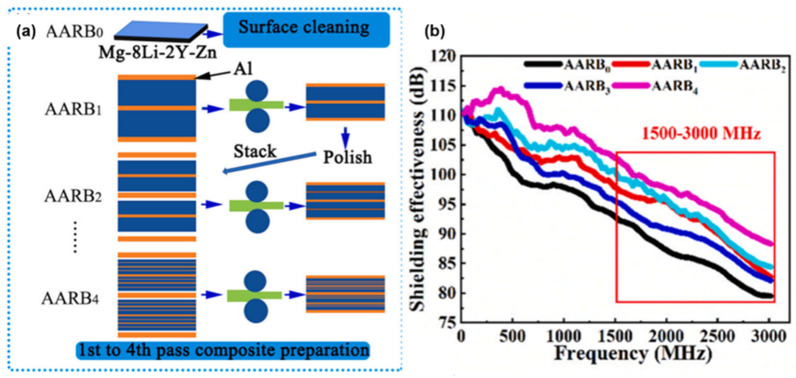
(**a**) Flowchart for the preparation of multilayer composites. (**b**) The effect of different number of AARB processing on the EMI SE [117].

In their study, Hu et al. prepared a multilayer composite material by stacking through the asymmetric accumulation rolling (AARB) process, in which Mg-8Li-2Y-Zn alloy acted as the core absorbing layer of the material, while pure aluminum was used as the reflecting layer; the preparation process is shown in Figure 16a. When electromagnetic waves enter the multilayer composite, they are greatly weakened by the synergistic effect of the absorber and reflector layers. In addition, the EMI SE of the composite increases gradually as the number of AARB processing increases. As shown in Figure 16b, the composite material processed four times with AARB achieved an EMI SE close to 115 dB at an electromagnetic wave frequency of 0.5 GHz [117].

**Figure 17:**
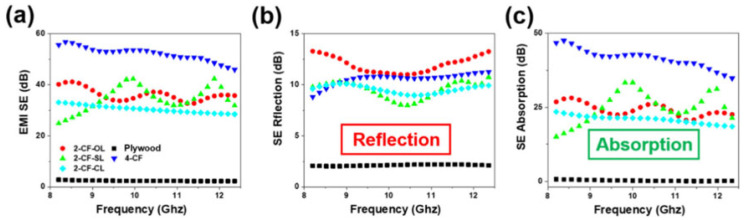
The effect of the position and number of CF sheets on EMI SE, (**a**) the overall electromagnetic shielding effectiveness of the material, (**b**) indicates the shielding effectiveness due to the reflection of electromagnetic waves by the material, and (**c**) indicates the shielding effectiveness due to the absorption of electromagnetic waves by the material. (4-CF, 2-CF-OL, 2-CF-CL, 2-CF-SL represent four CF sheets sandwiched in a five-layer veneer, two CF sheets sandwiched in two outer layers of a five-layer veneer, two CF sheets sandwiched in two core layers of a five-layer veneer, and two CF sheets placed in two surface layers of a five-layer veneer, respectively) [118].

Zou et al. fabricated a new electromagnetic shielding material by taking advantage of wood’s porous structure and anisotropy. They prepared an EMI shielding composite by impregnating CF sheets with wood veneer in epoxy resin and then combining them using the vacuum-assisted resin transfer molding technique. The EMI SE of the composites differed depending on the location and number of CF sheets, as shown in Figure 17. In addition, the composite achieved a flexural strength of 442.3 MPa and a tensile strength of 195.5 MPa, respectively. It also had good thermal conductivity and excellent surface hydrophobicity. This work provides a new direction to produce natural bio-based electromagnetic shielding materials [118].

Wang et al. fabricated a flexible MXene/FeCo alloy@carbon decorated carbon cloth (CC-FCM) composite that not only had good EMI SE but also had Joule heating and pressure sensing properties. In addition, the addition of a polydimethylsiloxane (PDMS) layer gave the carbon cloth a longer lifetime. The tensile strength of the composite material reached 403.2 MPa, and the EMI SE of a single piece of material reached 45.5 dB. The EMI SE of three pieces stacked together reached 80.5 dB, and after 1000 bends and 24 h of solution corrosion, its EMI SE could still maintain more than 90% of the original. In addition, the saturation temperature of the CC-FCM/PDMS composite can reach 105 °C by applying a voltage of 3.0 V, and the multilayer CC-FCM can also be used to make pressure sensors [119].

**Figure 18:**
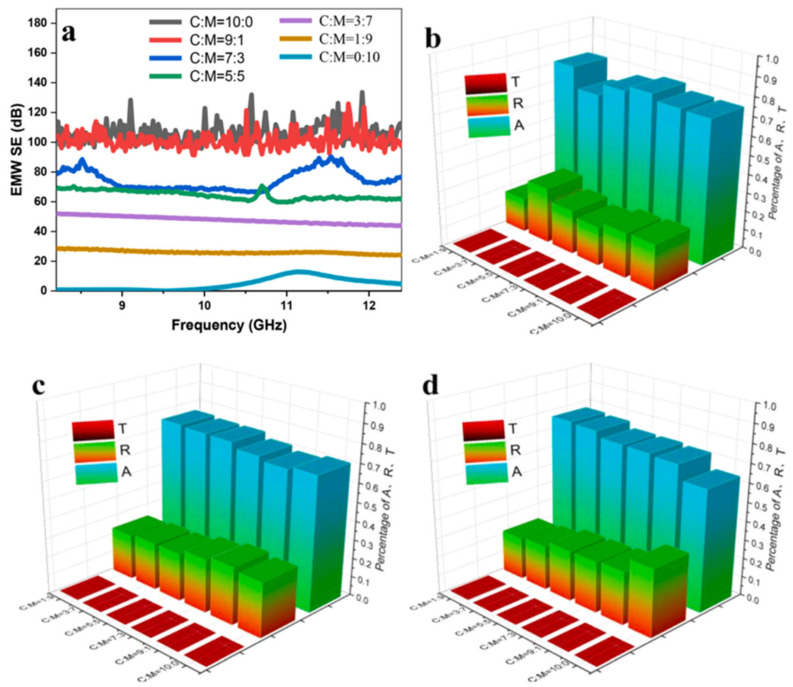
(**a**) EMI SE of the Ag microtubule (C)/BaFe_12_O_19_ (M) composite at the X-band. (**b**) T, R, and A coefficients of the composite at 8.2 GHz frequency. (**c**) T, R, and A coefficients of the composite at 10 GHz frequency. (**d**) T, R, and A coefficients of the composite at 12 GHz [120].

Recently, the practice of synthesizing nanomaterials has shifted from simple merging to the blending and accumulation of the basic properties of each material in a single composite material to better enhance the electromagnetic shielding effectiveness of the material. Absorptive electromagnetic shielding materials with excellent EMI SE were successfully prepared by combining hollow silver microtubes and magnetic barium ferrite nanoparticles by Gao et al. The parameters and impedance matching of the composite can be adjusted by changing the ratio of silver microtubes and barium ferrite nanoparticles. Due to the synergistic effect of the composites, the composites with a 9:1 ratio of silver microtubules to barium ferrite nanoparticles exhibited an excellent EMI SE of 100 dB in the X-band with an absorption coefficient of about 0.7 for both, as shown in Figure 18. In addition, the composite with a 3:7 ratio of silver microtubes and barium ferrite nanoparticles achieved an RL of −46.3 dB at the X-band with a thickness of only 3.0 mm [120].

**Figure 19:**
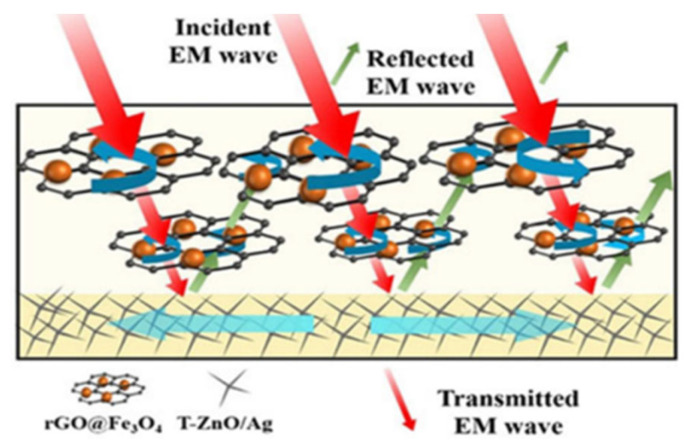
Schematic diagram of aqueous polyurethanes with gradient structures [122].

Biswas and colleagues demonstrated a multilayer structure that used an “absorption-multiple reflection-absorption” strategy to enhance SE_T_, where the top and bottom are the absorber layers and the middle is the reflector layer. The multi-walled carbon nanotubes MWCNT-MnO_2_ and RGO-Fe nanostructures in the PVdF matrix form the top and bottom absorber layers, and the MWCNTs in the PC/PVdF mixture form the reflector layer. These multilayer structures are 0.9 mm thick, exhibit an SE_T_ of 57 dB, and have an absorption of 92%. In addition, in another of their studies, they improved the impedance matching of the top layer using a functionalized MWCNT, thereby increasing the SE_T_ to 64 dB [121]. The “absorption–reflection–reabsorption process” was successfully demonstrated by creating a gradient layered structure within an aqueous polyurethane (WPU) film. This structure involves the distribution of rGO@Fe_3_O_4_ particles throughout the substrate and a thin bottom layer consisting of four needles of zinc oxide (T-ZnO)/Ag. The thick rGO@Fe_3_O_4_ layer within the PU substrate plays a key role in impedance matching and electromagnetic absorption. On the other hand, the thin T-ZnO/Ag layer at the bottom of the structure effectively re-reflects electromagnetic waves, thus further improving the overall absorption. Figure 19 presents a visualization of this concept. When the content of rGO@Fe_3_O_4_ was 5.7 vol.% and the sample thickness was 0.5 mm, the PU gradient structure composite exhibited an excellent SE_T_ of 87.2 dB, of which only 2.4 dB was SE_R_ [122].

Chang et al. proposed an ultra-efficient fiber-reinforced epoxy resin EMI shielding composite that could arbitrarily adjust the reflection and absorption ability of electromagnetic waves and had strong mechanical properties. The resin of the material was prepared by mixing EP, HHPA, and DMP-30 in the mass ratio of 100:79.6:5.4. In addition to the resin, there were three fabric compositions: CF (carbon fiber) layer at the bottom, a CNTs@ UF (urea-formaldehyde resin) layer in the middle, and Fe_3_O_4_@AF (amorphous fluoropolymers resin) layer at the top. The resin was infiltrated into the fibers by means of vacuum-assisted resin infusion. The composites were first preheated at 90 °C for 45 min at atmospheric pressure, cured at 90 °C for 4 h at 10 MPa, and then naturally cooled to room temperature to obtain fiber-reinforced epoxy composites. By adjusting the three main factors of suitable impedance matching of the incident layer, progressive conductive network, and effective thickness of each module, the composite exhibited an excellent average EMI SE of 78.6 dB and very low reflectance of 0.06 in the X-band. In addition, the material had excellent physical properties with a tensile strength of 283.1 MPa, which was due to the special layer structure of the material [123]. Zhao et al. fabricated MXene/(cellulose nanocrystals)CNC/(waterborne polyurethane)WPU (MCW-X) composite membranes with a special layered structure by vacuum-assisted alternating filtration, in which the outer layer consisted of MXene/WPU for the purpose of ensuring that the MXene/CNC was not oxidized. While an excellent conductive network was formed inside the composite film, the mechanical strength of the composite was further improved due to the interaction between the WPU and MXene nanosheets increasing the sliding between the MXene nanosheet layers. The modulus of elasticity and tensile strength of the material reached 3652.7 MPa and 52.2 MPa, respectively, and the MCW-4.5 composite with only 20 wt% CNC content achieved a conductivity of 606.1 S·cm^−1^ and an EMI SE of 54.2 dB (X-band) [124].

## 6. Conclusions and Future Outlook

Despite the high EMI SE and excellent mechanical properties of composite EMI shielding materials, they face challenges such as high production costs, complex processes, and limited mass production. This area still needs further research and exploration in order to expand the market scale and achieve higher benefits. Researchers have explored the frequency-dependent properties, morphological structure, and processing parameters of ICP and CPC to achieve enhanced EMI SE, where it has been demonstrated that they have great potential for EMI shielding.

The conductivity and structure of EMI shielding materials largely determine their shielding effectiveness. In structural designs such as porous, multilayer, and honeycomb, enhanced reflection loss can help improve the shielding effectiveness of the material. In addition, filler optimization is also an effective approach. The composite, dispersion, and orientation of nanofillers can have an impact on EMI SE. The content, morphology, surface modification, and dispersion distribution of conductive nano-fillers such as carbon, metal, and ferrite are regulated to enhance the absorption and reflection properties of the materials and enhance their synergistic effects to obtain higher EMI shielding efficiency.

Currently, many research efforts are focused on the development of composite materials that rely on absorption as the main mechanism to improve the EMI SE by using multiple reflections of electromagnetic waves within the material. However, the reflection coefficient of shielding materials is still relatively high, which poses a risk of secondary pollution to the environment. Therefore, there is an urgent need to promote composite shielding materials that exhibit high absorption, low reflection, or even no reflection, and thus can be widely used in various fields. In addition, EMI shielding foam materials have gained great interest in flexible electronics, aerospace, and smart devices due to their special structure and excellent thermal insulation properties. However, composite foams can exhibit degraded performance due to friction, extrusion, and stretching. At the same time, although porous composite EMI shielding foams can provide an excellent EMI SE, there are still some problems in terms of the high mechanical properties, lightweight characteristics, and tunability, so further research in these areas is necessary.

Finally, most EMI shielding fabrics used in industry today are still manufactured using chemical or electroplating processes, and these fabrics have great potential for applications in the flexible wearable sector. However, these manufacturing processes can cause significant environmental pollution because of the large amounts of heavy metals used in the manufacturing process. Therefore, addressing the challenge of environmental protection is critical to the development of shielding materials.

## Figures and Tables

**Table 1:** The σ and μ of other materials relative to copper [12].

Materials	Electrical Conductivity	Magnetic Permeability
Cu	1	1
Fe	0.17	50–1000
Ag	1.05	1
Au	0.7	50–1000
Al	0.1	1
Ni	0.2	1
Graphene	0.1	1.01
Carbon fiber	2.75 × 10^−2^	0.97
CNTs	0.1	1.01
Graphite	1.83 × 10^−2^	0.99

**Table 2:** EMI SE comparison of selected ICP materials.

Materials	d (mm)	Conductivity (S/cm)	EMI SE (dB)	Frequency (GHz)	Ref.
Ni@CNF paper	0.36	400	120	X-band	[26]
PPy/AgNW film	-	62.73	22.38	X-band	[22]
PANI/Sb_2_O_3_	-	-	(18–21)/(17.5–20.5)	X-band/ku-band	[23]
(CNT)/bamboo fiber/HDPE composites	-	11,000	49.6	X-band	[25]
PANI/Fe_3_O_4_	1	-	(15%PANI + 10%Fe_3_O_4_) 42	ku-band	[29]
		-	(15%PANI + 25%Fe_3_O_4_) 37.4	ku-band	
CNT/Fe_3_O_4_/Melamine-based carbon foam (MCF) functional material	3	0.8306	46.4	X-band	[30]
PDMS/CNT/PANI WA	-	0.186	26	X-band	[31]
(3D-CS)/SiCN	-	-	55	X-band	[32]
PVA/MXene multilayered film	0.027	0.716	44.4	X-band	[33]
MXene/PEDOT:PSS hybrid film	0.0066	675.2	40.5	X-band/ku-band	[34]
MXene/wood (WP-MXene/Delignified wood)	-	-	43.4	X-band	[35]
MWCNT-MXene@Cotton Fiber (MWMC)	1	-	40.6	X-band	[36]
Ti_3_C_2_T_x_/TiO_2_ heterostructured	-	-	35.1	ku-band	[37]
			21	X-band	
Ti_3_C_2_T_x_/WVO_2_	-	-	42.8	X-band	[38]
AgNF/MXene/AgNW (AMA)	0.026	500<	71	X-band	[39]

**Table 3:** EMI SE comparison of selected CPC materials.

Materials	d (mm)	Filler Concentration	Conductivity (S/cm)	EMI SE (dB)	Frequency (GHz)	Ref.
Thermally reduced graphene oxide (TGRO)/ultrahigh molecular weight polyethylene (UHMWPE)	-	0.660 vol.%	0.34	28.3–32.4	X-band	[58]
Reduced graphene oxide (RGO)/PS	2.5	3.47 vol.%	0.44	45.1	X-band	[59]
PC/ -acrylonitrile (SAN) 60/40 MWCNTs	10	1 wt%	0.083	25–29	X-band	[64]
SR/CNF wafers	0.75	2 wt%	-	38.95	2–18.4	[68]
Graphene foam (GF)/poly(3,4 ethylenedioxythiophene):poly(styrenesulfonate) (PEDOT:PSS)	1.5	0.0762 g/cm^3^	43.2	91.9	X-band	[75]
Graphene/epoxy		8.8 vol.%	0.1	21	X-band	[77]
Acrylonitrile-butadiene-styrene (ABS)/CNT	-	3 wt%	0.1	16	ku-band	[80]
Multifunctional CNT/polylactic acid (PLA) film	4	4 wt%	-	68	12.3	[81]
PDMS/expanded microspheres (EM)/CNT	-	EM/50 vol.%; CNT/1.74 vol.%	0.66	43	X-band	[15]
PVdF/MWCNT/GN	2	4.5 vol.% (MWCNT:GN = 1:1)	0.199–0.22	28.5	X-band	[82]
Ni@MWCNT/MWCNTs	-	Ni@MWCNTs/10 vol.%; MWCNTs/1.74 vol.%	-	77.2	X-band	[83]
Flexible and porous Cu/poly (L-lactic acid) (PLLA) fiber	0.015	-	9472	39.59/39.96	H-band/Ku-band	[84]
Isomeric polypropylene (iPP)/polyethylene-1-octene (POE)	1.2	3.0 vol.%	0.00003	25	X-band	[85]
PVDF/ethylene-α-octene block copolymer (OBC)/MWCNT	2	MWCNT/2.7 vol.%	-	34	X-band	[86]
PA6 @NiM/MWCNT/PS	-	PA6 @NiM/10 wt%; MWCNT/7 wt%	0.64	46.9	X-band	[88]

## Data Availability

Not applicable.
